# Imparting scalephobicity with rational microtexturing of soft materials

**DOI:** 10.1126/sciadv.adj0324

**Published:** 2023-12-20

**Authors:** Julian Schmid, Tobias Armstrong, Fabian J. Dickhardt, SK Rameez Iqbal, Thomas M. Schutzius

**Affiliations:** ^1^ Laboratory for Multiphase Thermofluidics and Surface Nanoengineering, Department of Mechanical and Process Engineering, ETH Zurich, Sonneggstrasse 3, CH-8092 Zurich, Switzerland.; ^2^ Department of Mechanical Engineering, University of California, Berkeley, CA 94720, USA.

## Abstract

Crystallization fouling, a process where scale forms on surfaces, is widespread in nature and technology, negatively affecting energy and water industries. Despite the effort, rationally designed surfaces that are intrinsically resistant to it remain elusive, due in part to a lack of understanding of how microfoulants deposit and adhere in dynamic aqueous environments. Here, we show that rational tuning of coating compliance and wettability works synergistically with microtexture to enhance microfoulant repellency, characterized by low adhesion and high removal efficiency of numerous individual microparticles and tenacious crystallites in a flowing water environment. We study the microfoulant interfacial dynamics in situ using a micro-scanning fluid dynamic gauge system, elucidate the removal mechanisms, and rationalize the behavior with a shear adhesive moment model. We then demonstrate a rationally developed coating that can remove 98% of deposits under shear flow conditions, 66% better than rigid substrates.

## Teaser

Microfoulant removal can be enhanced through rational engineering of soft materials at the microscale.

## INTRODUCTION

Water and energy are interconnected resources, with water necessary for energy production and energy required for water transportation, treatment, and desalination. However, the finite nature of both resources and the growing global challenges, such as climate change and population growth, place them under increasing stress (*1*). Globally, strategic objectives are being established to counteract the stress by optimizing the water use efficiency in energy production and improving the energy efficiency in water treatment (*2*–*4*). Efficiency gains can be achieved by various technical means, e.g., reducing heat waste by recovering the dissipated energy in power generation with innovative thermoelectric materials (*5*), reusing water harvested in cooling towers to reduce water consumption in thermoelectric power generation (*6*, *7*), decreasing losses in desalination processes, and improving heat exchanger technology using membrane engineering and advanced interfacial materials to overcome the severe fouling problems (*3*, *8*, *9*). Fouling on membranes and heat exchanger surfaces can substantially degrade the ability to separate and convert the energy of water and energy systems efficiently. Crystallization fouling or “scaling” is an important subset of fouling that results from the precipitation of retrograde soluble salts such as calcium carbonate or calcium sulfate. Such scaling salts are common components of fouling deposits—termed “scale”—in industrial heat exchangers and on membranes, which substantially inhibit heat transfer and flow performance. Scale can lead to an enormous energy loss (at least 2% of the total world energy production per year) due to efficiency reduction in heat transfer and flow performance (*10*).

Mitigation, prevention, and removal of the scale are currently addressed primarily by active methods (*11*, *12*). However, the effectiveness, applicability, and scalability of these techniques are limited (*13*–*15*). Researchers have investigated passive methods for repelling scale such as surface engineering, interfacial materials, and coatings, which can control the interaction between the scale and surface (*16*, *17*). These methods are considered attractive alternatives to achieve sustainability and cost-efficiency. Previous work on surface engineering has focused on developing rigid antifouling surfaces by altering the surface energy of the materials (*18*–*21*) and reducing the roughness (*22*) to eliminate fouling. There has been a growing interest in interfacial materials and compliant coatings that aim to enhance the antifouling properties by using the materials’s inherent barriers (*23*–*34*).

The assessment of the antifouling properties of the surfaces mentioned above has primarily focused on nucleation tests in beakers, evaporation tests, or flow setups, with less attention paid to the importance of accessing adhesion and observing removal at the microscale in situ. Most measurements that have been conducted to access the microscale adhesion and removal interaction focused on ex situ methods. In these approaches, crystallites were either grown in situ, and their adhesion was measured ex situ (*35*), or they were attached to a force measurement device (*20*, *21*, *36*–*38*), and the adhesion was evaluated in submerged conditions. In cases where the crystallites were grown in situ on surfaces, and the adhesion was evaluated in submerged conditions (*20*, *39*, *40*), the shear flow removal was not assessed using optical image analysis. Flow setups (*33*, *34*, *41*, *42*) often suffer from a lack of optical access. To quantify fouling, they use pressure drop, fouling resistance, or foulant mass measurements. Therefore, these tests offer a macroscopic perspective and do not provide detailed insights into the microscale physics of foulant removal. In cases where researchers studied fouling deposit removal in a flow chamber, they only imaged at low magnification (macroscale) or only provided a few measurements which are insufficient for statistical interpretation (*29*, *32*, *43*). Hence, the existing methods are not able to investigate the mechanisms of passive crystallite removal. We define passive here to mean crystallites that are removed from the substrate surface by the prevailing flow conditions. Revealing these mechanisms is crucial to the design of scalephobic surfaces. In addition, the current understanding of crystallite-surface-water interactions is poor. Thus, there is a clear demand to study the removal of microscale crystallites in situ, which is needed for the rational design and development of compliant antifouling surfaces.

Here, guided by fluidic and adhesion theories, we develop a methodology to study the physics of microfoulant adhesion and removal in situ on engineered surfaces, focusing on the intertwined effects of shear flow, material compliance, wettability, and surface microtexture on microfoulant adhesion and removal across a range of foulant types and sizes. For this purpose, we created a micro-scanning fluid dynamic gauge (μ-sFDG) setup, which provides the necessary spatial and temporal resolution paired with critical shear flow quantification to observe in situ passive shear removal of real scale (calcium carbonate) and model microfoulants (polystyrene particles) from various substrates. We reveal three underlying mechanisms of microfoulant removal from compliant materials, i.e., gliding, rolling, and shedding, the last of which we rationalize with a theoretical adhesive-shear-moment balance model. Guided by this model, we then impart surface microtexture and show enhanced removal efficiencies—leveraging the shedding mechanism—to microfoulants on soft coatings. Last, we use this knowledge to design a microtextured compliant coating with passive scale-shedding properties and test its scalephobicity under laminar and turbulent flow conditions. We expect that this work will improve our understanding of crystallization and particulate fouling and can assist in the design of antifouling surfaces, especially compliant interfacial materials, which are needed to address the challenges of the water-energy nexus.

## RESULTS

We take inspiration from nature’s exceptional examples of superwettability and transport systems (*44*, *45*), which can incorporate foulant repellency, and previous research on compliant repellent substrates (*46*) to study the dynamics of crystallite-surface-water interactions. First, we quantify the microfoulant removal from substrates with varying compliance, Young’s modulus, *E*, ranging from 12 kPa to 70 GPa, and varying wettability (see Table 1 for details on the nomenclature, treatment, compliance, and wettability of the tested substrates). To remove calcium carbonate crystallites—with sizes ranging from 5 to 15 μm—from the substrates, we impose a tunable laminar water shear flow and simultaneously visualize the removal with an inverted microscope. This shear flow is generated by pumping water through a glass capillary that is brought near the test surface and the water that drains between the bottom of the capillary and substrate generates the shear stress. We apply flow rates ranging from $\dot{V}$ = 7 to 103 ml min^−1^, resulting in bulk flow velocities of $\bar{v}$ = 0.2 to 6.0 m s^−1^ for a capillary-substrate separation distance of 80 μm. With this methodology, we can quantify in situ the passive shear-driven removal process.

**Table 1.** Nomenclature and material properties for compliant and rigid substrates. A detailed description of the Young’s modulus measurement and contact angle measurements are described in Materials and Methods. Young’s modulus values for Glass and PFDTES are literature values (*81*). –, indicates that the value is too small to be measured.

Nomenclature	Material/treatment	Young’s modulus E	Wettability
Glass	Cleaned glass	70 GPa	$\theta_{a}^{*}$ = 41.2° ± 2.9°
			$\theta_{r}^{*}$ = 13.5° ± 0.4°
PFDTES	Cleaned glass + 1H,1H,2H,2H-perfluorodecyltriethoxysilane silanization	70 GPa	$\theta_{a}^{*}$ = 117.3° ± 0.7°
			$\theta_{r}^{*}$ = 96.0° ± 1.0°
PDMS 2:1	Cleaned glass + Sylgard 184 coating; ratio 2:1	527.5 ± 2.1 kPa	$\theta_{a}^{*}$ = 127.8° ± 1.0°
			$\theta_{r}^{*}$ = 70.7° ± 1.8°
PDMS 10:1	Cleaned glass + Sylgard 184 coating; ratio 10:1	1112 ± 36 kPa	$\theta_{a}^{*}$ = 120.4° ± 1.0°
			$\theta_{r}^{*}$ = 66.2° ± 1.4°
PDMS 30:1	Cleaned glass + Sylgard 184 coating; ratio 30:1	108.6 ± 6.1 kPa	$\theta_{a}^{*}$ = 129.5° ± 0.8°
			$\theta_{r}^{*}$ = 23.9° ± 0.2°
PDMS 50:1	Cleaned glass + Sylgard 184 coating; ratio 50:1	11.6 ± 1.8 kPa	$\theta_{a}^{*}$ = 138.6° ± 0.5°
			$\theta_{r}^{*}$ = –
CY52-256	Cleaned glass + CY52-256 coating; ratio 5:6	11.1 ± 0.5 kPa	$\theta_{a}^{*}$ = 120.0° ± 0.5°
			$\theta_{r}^{*}$ = 70.9° ± 1.0°
PEG-DA 10	Cleaned glass + 10 wt % poly(ethylene glycol) diacrylate coating	79.5 ± 0.7 kPa	$\theta_{a}^{*}$ = 43.2° ± 1.4°
			$\theta_{r}^{*}$ = –
PEG-DA 50	Cleaned glass + 50 wt % poly(ethylene glycol) diacrylate coating	6236 ± 267 kPa	$\theta_{a}^{*}$ = 37.2° ± 1.7°
			$\theta_{r}^{*}$ = –

**Fig. 1.**
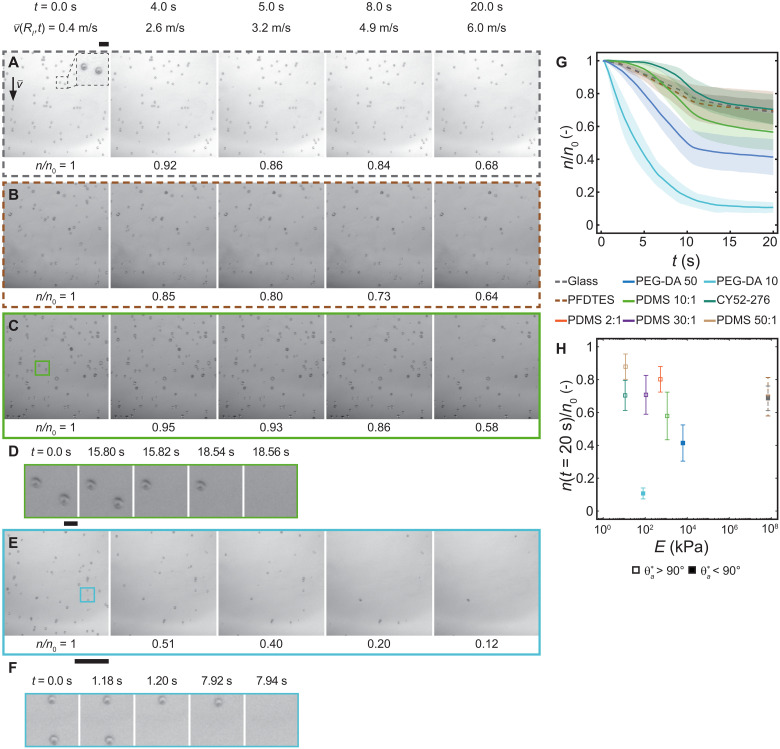
Microfoulant dynamics under shear-driven water flow. Bottom-view image sequence showing calcium carbonate crystallites on (**A**) uncoated glass, (**B**) 1H,1H,2H,2H-perfluorodecyltriethoxysilane (PFDTES)–coated glass, (**C**) polydimethylsiloxane (PDMS 10:1)–coated glass (coating thickness, δ ≈ 10 μm), and (**E**) poly(ethylene glycol) diacrylate (PEG-DA 10)–coated glass (δ ≈ 10 μm) immersed in water and subjected to a shear flow (starting at *t* = 0 s, the flow rate increases from 7 to 103 ml/min in a channel of 80-μm height, resulting in a bulk velocity, $\bar{v}$ = 0.2 to 6 m s^−1^). The inset image reveals the crystallite diameter to be approximately 5 to 15 μm. Zoom images showing the removal of single crystallites from the compliant substrates (**D**) PDMS 10:1 and (**F**) PEG-DA 10. We define the number of visible crystallites on the surface to be *n*, and its initial value, *n*_0_. (**G**) Temporal evolution of *n*/*n*_0_ for various coatings on glass substrates. Lines representing the mean values and shaded regions are the SD for *e* ≥ 9 experiments on *N* = 3 independent samples. (**H**) Influence of stiffness and wettability on *n*(*t* = 20 s)/*n*_0_ for various coatings on glass substrates. Scale bars, (A) to (C) and (E) 100 μm; inset: (A) 10 μm and (D) and (F) 10 μm.

Figure 1A shows an image sequence of a rigid glass substrate (Glass), which has undergone crystallization fouling (fig. S1) and was then subjected to a water shear flow (see Materials and Methods). From time zero, *t* = 0 s, to *t* = 10 s, we linearly increase the water flow rate from $\dot{V}$ = 7 to 103 ml min^−1^, resulting in $\bar{v}$ increasing from 0.2 to 6 m s^−1^, and maintain a constant flow rate until *t* = 20 s (see fig. S2). During this, we observe how the number of crystallites on the surface, *n*, changes relative to the initial value, *n*_0_, which for Glass goes from *n(t)*/*n*_0_ = 1.0 to 0.68 (see fig. S3 for details on postprocessing of the images and crystallite detection). Figure 1 (B and C) shows the same experiment, except that the glass is now treated with a fluorosilane (PFDTES, 1H,1H,2H,2H-perfluorodecyltriethoxysilane) and a soft silicone [polydimethylsiloxane 10:1 (PDMS 10:1)] coating, respectively, rendering them hydrophobic (see movie S1). In the case of the PFDTES-coated rigid glass, we see an unexpected similarity in the number of crystallites that are removed compared to the Glass sample, despite their substantially different surface chemistry [*n*(*t* = 20 s)/*n*_0_ = 0.64]. In contrast, for the soft PDMS 10:1–coated glass, we see that 10 and 6% more crystallites are removed compared to Glass and PFDTES, respectively, a notable increase, and that the removal process once it is initiated is rapid (Fig. 1D). Figure 1E shows the final case, where the glass is coated with another soft coating [poly(ethylene glycol) diacrylate 10 (PEG-DA 10)], which is hydrophilic instead of hydrophobic, and we see that 88% of the crystallites are removed and that the removal process for an individual crystallite is also rapid (Fig. 1F; see movie S2). Figure 1G shows a plot of *n*/*n*_0_ versus *t* for these four sample types and an additional silicone coating (CY52-276). CY52-276 is more compliant than PDMS 10:1 and PEG-DA 50 is stiffer than PEG-DA 10 due to its higher polymer content (see fig. S4). Figure 1H shows a plot of *n*(*t* = 20 s)/*n*_0_ versus *E* for all of the previously mentioned coatings and additionally PDMS coatings with varying curing ratios from 2:1 to 50:1 (fig. S5).

From Fig. 1H, we see that for the hydrophobic coatings, decreasing *E* first results in lower values of *n*(*t* = 20 s)/*n*_0_, but then they increase again. For the hydrophilic coatings, we see a continuous decrease in *n*(*t* = 20 s)/*n*_0_ for decreasing *E*. We can rationalize the results for the compliant coatings using Johnson-Kendall-Roberts (JKR) theory (*47*–*49*). Assuming that the microfoulant is spherical and rigid and that the coating is compliant, the microfoulant is expected to indent into the coating, and the microfoulant-coating contact radius should scale as *a* ∝ 1/*E*^1/3^, where *E* is the Young’s modulus of the coating. Therefore, *a* should increase with decreasing *E* for the microfoulants, increasing the contact area and reducing the microfoulant surface area exposed to the water shear flow, making them more difficult to remove. However, we observe the opposite trend for the PEG-DA coatings, where decreasing *E* results in lower values of *n*(*t* = 20 s)/*n*_0_. This highlights the intricate nature of the substrate-crystallite interaction, demonstrating that depending on substrate wettability, softer substrates are not always superior, which we explore in the following with model microfoulants.

Although the composition and wettability differ between all the substrates, we observe that individual crystallite removal events are similarly rapid for most of them (20 ms or less). Only the removal events on PDMS 2:1 (fig. S5 and movie S3) are different. The crystallites appear to “glide” along the surface, which we attribute to a lubrication layer caused by the uncross-linked PDMS. The PEG-DA 10 coating sheds crystallites at considerably lower values of $\bar{v}$ compared to the other coatings. This has substantial implications for antifouling or scalephobic materials as it allows us to remove the crystallites before they build up into tenacious scale layers.

**Fig. 2.**
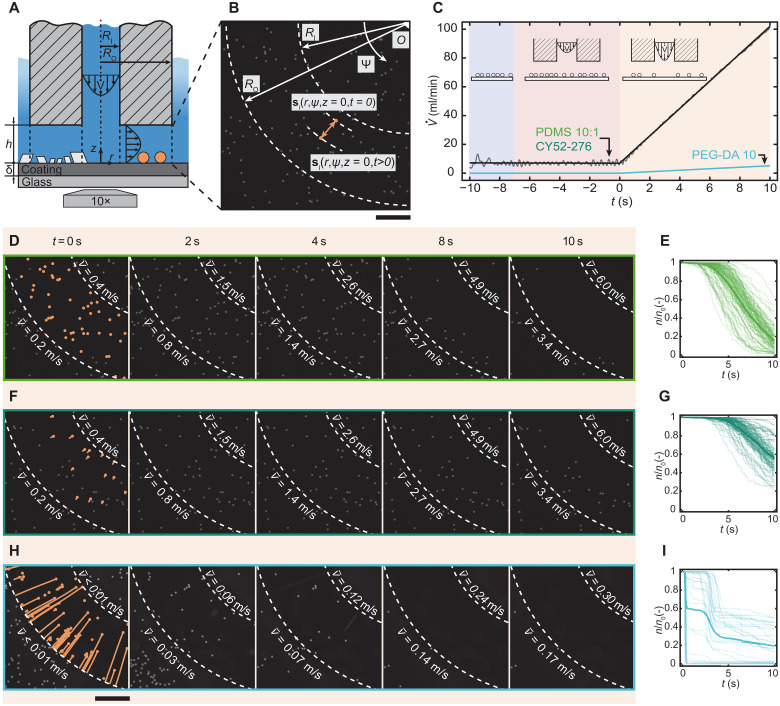
μ-sFDG enables insight into compliance-induced microfoulant removal. (**A**) Schematic of the μ-sFDG test section: The glass capillary is connected to a fluidic system, which provides a radial laminar Poiseuille flow in the channel (*h* = 80 μm; *Re*_gap_ = $2\rho \dot{V}/\mu (2\pi R_{I}+h)$ < 1400) between the nozzle and tested material. We define the point of a given particle, “*i*”, on the surface beneath the nozzle as a function of time, *t*, as **s**_***i***_(*r*,ψ,*z* = 0,*t*) = *s*_*r*,*i*_(*t*)***e***_***r***_ + *s*_ψ,*i*_(*t*)***e***_**ψ**_, (*r* ∈ (*R*_I_, *R*_O_) at time zero and *i* ≤ *n*_0_). (**B**) Epifluorescent image of the coating surface (*z* = 0), fouled with polystyrene microparticles (diameter *D* = 10 μm). Dashed lines show inner, *R*_I_ = 560 μm, and outer radii, *R*_O_ = 1000 μm, of the nozzle. (**C**) Experimental procedure showing the ramp-up of the volume flow, $\dot{V}$ versus *t*. The gray line represents the mean value of $\dot{V}$ of five experiments, and black and blue lines represent fitted data. Background colors indicate the different stages of the experiment: blue, nozzle at position *h* = 2000 μm; red, approach *h* = 80 μm; orange, linear ramp up of $\dot{V}$. Bottom-view image sequences showing the position of the microfoulants beneath the nozzle and bulk flow velocities $\bar{v}$, at *r* = *R*_I_ and *R*_O_, on (**D**) PDMS 10:1, (**F**) CY52-276, and (**H**) PEG-DA 10 samples (δ ≈ 10 μm). The image at time zero shows the projected (*r*-ψ plane, *z* = 0) trajectories of removed microfoulants (orange). We define the initial number of visible microfoulants on the surface beneath the nozzle to be *n*_0_(*r*,ψ,*z* = 0,*t* = 0 *s*), [*r* ∈ (*R*_I_, *R*_O_)], and its temporal value to be *n*(*r*,ψ,*z * = 0,*t*) ⊂ *n*_0_. (**E**, **G**, and **I**), *n*/*n*_0_ versus *t* for the different coatings. Bold lines represent mean value and transparent lines are individual experiments for *e* ≥ 28 experiments on *N* = 5 independent samples. Scale bars, (B), (D), (F), and (H) 200 μm.

To understand the mechanisms responsible for enhanced repellency toward scale on compliant coatings, we replace the complex scale crystallites—which can vary in size and polymorphs (*36*)—with comparably sized spherical polystyrene microparticles (fluorescent; diameter *D* = 10 μm). We refer to these particles as microfoulants from here onward and use them to study the effect of water shear on their displacement and removal across a range of foulant sizes and substrate properties including Young’s modulus, wettability, and thickness. Besides crystallization fouling, particulate fouling, which we obtain by settling of microfoulants on the coating, represents another important subset of fouling (*50*). Figure 2A shows a schematic of the experimental setup that we used to observe and quantify the microfoulant dynamics, namely, a micro-scanning fluid dynamic gauge system (μ-sFDG). This setup allowed us to study interfacial dynamics, in our case the adhesion and removal of foulants, in situ with the necessary spatial and temporal resolution comparable to previous work where the stable underwater superhydrophobic state was investigated (*51*). (A detailed description of the setup, its scanning function, and the experimental protocol is provided in Materials and Methods and fig. S6.) Briefly, we impose a tunable laminar water shear flow—the Reynolds number is *Re*_gap_ = $2\rho \dot{V}/[\mu (2\pi R_{I}+h)]$ < 1400—by pumping water through a cylindrical-shaped glass capillary nozzle that is brought near to the test surface. By using a nozzle-substrate separation distance, *h*, that is substantially less than the nozzle wall thickness, *R*_o_ − *R*_I_, the water is forced to drain between the two interfaces and generates a shear stress that acts on the microfoulant. (A detailed description of estimating the shear stress is given in the caption of fig. S7.)

Figure 2B shows the bottom view of the coating fouled with microfoulants, visualized with an inverted fluorescence microscope (see Materials and Methods). We can define the position vector of a given particle, “*i*”, on the surface beneath the nozzle as a function of time, *t*, as **s**_***i***_(*r*,ψ,*z =* 0,*t*) = *s*_*r*,*i*_(*t*)***e***_***r***_ + *s*_ψ,*i*_(*t*)***e***_**ψ**_, where *r* ∈ (*R*_I_, *R*_o_) at time zero and *i* ≤ *n*_0_, and we track the position **s** and localize the removal events spatially and temporally during the experiment. We define removal as substantial displacement of the foulant position either in the *z* direction, meaning shedding and therefore the particle is no longer detectable on the surface, or substantial displacement in the *r*-ψ plane (Δ***s***_***i***_ > 1.5*D* and ***s***_***i***_ > *R*_o_). Using the developed setup, we obtain for each experiment approximately 20 to 120 individual microfoulant removal events. By using the scanning function of the μ-sFDG, we can evaluate up to 1200 removal events per sample. This can be achieved by precisely replicating the same values of *h* (using the piezo nano-micro stages) and $\dot{V}$ for each experiment (Fig. 2C). From *t* = −10 s to *t* = 0 s, we keep $\dot{V}$ constant and move the nozzle toward the substrate to obtain stable flow conditions before we linearly increase $\dot{V}$ and therefore $\bar{v}$. Figure 2 (D, F, and H) shows epifluorescent image sequences of fouled PDMS 10:1, CY52-276, and PEG-DA 10 subjected to increasing $\bar{v}$, respectively, and Fig. 2 (E, G, and I) shows the corresponding plots of *n*/*n*_0_ versus *t*. The images at time zero show the projected (*r*-ψ plane at *z* = 0) position trajectories of the microfoulants when they are in contact with the coating surface in orange. For Fig. 2 (D and F), the relatively short projected trajectories indicate that the microfoulants were initially displaced relatively little by the flow before rapidly detaching from the substrate surfaces.

We observe for PDMS 10:1 and CY52-276, similar to the crystallites, that the microfoulants undergo sudden and rapid movement after a small to medium displacement from their initial position in the *r*-ψ plane, a process that we term “shedding” (compare movies S1 and S4). This is counterintuitive as literature suggests that rolling removal is the primary removal mechanism for spherical particles (*52*, *53*). On PEG-DA 10, we see that the first microfoulants begin to move at the moment the shear flow starts. The motion is slower and the microfoulant appears to “glide” along the surface, which is what we term the mechanism (gliding), and attribute it to a thin water lubrication layer. At later times and higher flow velocities, the removal dynamics are similar to detachment. We attribute this to surface defects and a breakdown of the lubrication layer (Video S4). In contrast, the removal mechanism for the crystallites on PEG-DA 10 resembled only shedding. For PDMS 10:1 and CY52-276, we observe that the final fraction of microfoulants remaining on the coating surfaces is *n*/*n*_0_ ≈ 0.16 and 0.56, respectively. The results imply that the value of $\bar{v}$ required for removal is higher on CY52-276, which is also consistent with our crystallite results. For PEG-DA 10, we see that the final value of *n*/*n*_0_ is 0.19, a substantially lower value, similar to the crystallite experiments. This result is noteworthy, considering that the maximum value of $\bar{v}$ is an order of magnitude lower than that used for the other two soft coatings. Looking at the removal behavior we observe that there is an initial period of particle removal (gliding) as well as a later one (shedding). As a result, the mean value of *n*(*t*)/*n*_0_ shows a step-like behavior. Adhesion theory (*54*) and studies on ice (*55*, *56*) and hydrate fouling (*57*) have found that the force required to remove a rigid object or foulant from compliant materials tends to decrease as the material thickness increases. In our material thickness variation experiments (fig. S7), we observe a similar trend for PEG-DA 10. However, for the CY52-276 coating, we find an inverse relationship, and for PDMS 10:1, a non-linear relationship with the intermediate coating thickness yields the lowest hydrodynamic force necessary for removal and highest removal performance. We attribute the deviation from the existing literature to the fact that the microfoulants are small, comparable to, or much smaller than the thickness of the coating (*58*), whereas the length scale of ice and hydrate foulants exceed the thickness of the coating (*55*–*57*).

**Fig. 3.**
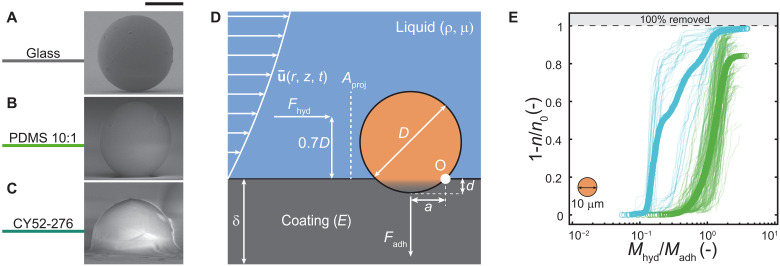
Compliance and wettability alter microfoulant adhesion in shear flow environments. Side-view micrographs showing the contact behavior of a foulant (*D* = 20 μm) on (**A**) glass, (**B**) PDMS 10:1, and (**C**) CY52-276 in a vacuum environment. For PEG-DA 10, we are not able to show micrographs as the sample would not survive the vacuum in the SEM or need to be freeze-dried, which would change the contact behavior. (**D**) Schematic of theoretical moment analysis at point O of the removal mechanism on compliant material in a shearwater flow environment. The shear force working on the microfoulant surface can be expressed as an effective hydrodynamic force *F*_hyd_ acting on the foulant at a lever length distance, 0.7*D*, from point O, generating the removal moment *M*_hyd_. Similarly, the adhesion moment *M*_adh_ holding the microfoulant on the coating can be expressed as the adhesion force *F*_adh_ acting at a lever length distance *a*, which is equal to the contact radius. (**E**) Removal efficiency (1 − *n*/*n*_0_) versus momentum ratio (*M*_hyd_/*M*_adh_) for PEG-DA 10 (blue, *E* = 79.5 kPa, *W*_adh_ ≈ 0.001 J/m^2^) (*62*) and PDMS 10:1 (green, *E* = 1112.2 kPa, *W*_adh _≈ 0.041 J/m^2^), coating thickness δ ≈ 100 μm (see Materials and Methods for material characterization). Circles represent the overall performance and semitransparent lines represent individual experiments, for *e* ≥ 32 experiments on *N* = 5 independent samples. Scale bars, (A) to (C) 10 μm.

To understand the shedding and gliding removal behavior and differences in the performance of the compliant coatings, we need to understand the microfoulant-coating interaction. Figure 3A shows a side-view micrograph of a microfoulant in contact with a rigid glass substrate. The image reveals that the microfoulant-substrate contact radius, *a*, is relatively small compared to the microfoulant diameter, *D*. In contrast, Fig. 3 (B and C) shows a microfoulant on the compliant coatings PDMS 10:1 and CY52-276, respectively, and for both cases, the contact radius increases compared to glass. The microfoulant on PDMS 10:1 penetrates much less into the coating than the microfoulant on the CY52-276 coating. We determine the value of *a* ≈ 2.1 μm on PDMS 10:1 using JKR theory and SEM imaging. We attribute this deeper microfoulant penetration on the softer CY52-276 to indentation, a mechanism driven by capillary effects that cannot be described by JKR theory (*59*). JKR theory is valid when *a* is much larger than the elastocapillary length of the coating, *l* = γ_SV_/*E*, where γ_SV_ is the solid-vapor surface energy of the coating, and for CY52-276 we calculate *l* ≈ 2.7 μm, which is only slightly smaller than the measured contact radius *a *≈ 3.8 μm (γ_SV_ = 31 mN m^−1^ (*60*), *E* = 11.1 kPa). All of the imaging and calculations done here were for a microfoulant on a surface in a gas environment; however, we assume that this behavior still persists in an underwater environment too. Specifically, this observed indentation effect is likely responsible for the reduced microfoulant removal efficiency of CY52-276 coating compared to PDMS 10:1 (see Fig. 2G).

Figure 3D shows a schematic of the microfoulant-coating–shear flow interaction and the relevant forces and dimensions. On the basis of our experimental findings and understanding of the microfoulant-coating interaction, and building on and further extending a previous analysis (*61*), we analyze the shear-driven and adhesive moments acting on the microfoulant around a point *O* to elucidate the underlying mechanism governing the dynamics of microfoulant removal. This analysis accounts for the effects of foulant size, coating properties, applied shear flow, and contact behavior. Point *O* is the point where the microfoulant begins to rotate around once the hydrodynamic moment, *M*_hyd_, is sufficiently large to overcome the adhesive moment, *M*_adh_, initiating its motion and ultimately causing it to detach. Assuming JKR theory, we can define the adhesive moment as the product of its force, *F*_adh_ = (3/2)π*W*_adh_*D*, and the lever length, which is the contact radius, *a*, where *W*_adh_ is the work of adhesion. We obtain values for *W*_adh_ from our adhesion experiments in water or literature (see Materials and Methods) (*62*) and we determine the contact radius from *a* = [9π*W*_adh_(1 − *v*^2^)/2*E*]^1/3^(*D*/2)^2/3^ JKR theory (*47*, *49*, *63*), where *v* is the coating’s Poisson ratio (for incompressible material, *v* = 0.5). Similarly, if we assume that the microfoulant does not penetrate significantly into the coating (*d ≪ D*), then we can define the hydrodynamic moment as the product of its force, *F*_hyd_, and the lever length, 0.7*D* (*64*, *65*). Depending on the microfoulant Reynolds number, $Re_{D}=\rho \bar{u}D/\mu$, we need to distinguish between Stokes drag (*Re*_D_ < 0.1) and inertial drag (*Re*_D_ ≥ 0.1). For Stokes drag, we obtain, $F_{\mathrm{hyd}}=3\pi \mu \bar{u}Df$, where μ is the dynamic viscosity of the water and *f* is a correction factor (1.7009), which accounts for the presence of the wall (*65*). Inertial drag can be defined as $F_{\mathrm{hyd}}=\frac{1}{2}\rho_{f}C_{D}A_{\mathrm{proj}}\;{\bar{u}}^{2}$, where ρ_f_ is the water density; *C*_D_ the drag coefficient; $A_{\mathrm{proj}}\approx \left[\pi {\left(D/2\right)}^{2}-\frac{4}{3}ad\right]$ is the projected area of the foulant; and $\bar{u}$ is the mean flow velocity near the foulant (see note S1 for details on *C*_D_ and $\bar{u}$ calculation). Therefore, the resulting moment ratio can be described as

$$\frac{M_{\mathrm{hyd}}}{M_{\mathrm{adh}}}=\left\{ \begin{array}{c} \frac{21\mu \bar{u}Df}{10W_{\mathrm{adh}}a},Re_{D}<0.1\\ \frac{7\rho_{f}C_{D}A_{\mathrm{proj}}{\bar{u}}^{2}}{20\pi W_{\mathrm{adh}}a},Re_{D}\geq 0.1 \end{array}\right.$$ (1)

Note that an additional factor of 2/3 included in the moment analysis represents the maximum possible adhesion moment that the microfoulant can experience, which was determined in previous work (*61*). In Fig. 3E, we plot the removal efficiency, 1 − *n*/*n*_0_, versus *M*_hyd_/*M*_adh_ from Eq. 1 for PEG-DA 10 and PDMS 10:1, where each semitransparent line represents an individual experiment and the circles represent the overall performance.

We find that the theoretical order of magnitude analysis yields a good fit for the dynamics of microfoulants on PDMS 10:1, both its trend and that a substantial fraction of the microfoulants is removed for values of *M*_hyd_/*M*_adh_ near unity. The validation of these findings is further supported by the observations presented in Fig. 2D and movie S4 where we observe shedding from the surface. For PEG-DA 10, if we examine the individual experiments (transparent lines in Fig. 3E), then we can see two types of removal behavior: one whose onset of microfoulant removal occurs at *M*_hyd_/*M*_adh_ ≈ 0.1, indicating that we are overestimating the adhesion, and another at *M*_hyd_/*M*_adh_ ≈ 0.5, which is more consistent with the behavior of PDMS 10:1 and our model. The former observation is consistent with the gliding behavior we saw in Fig. 2H and movie S4, where it is evident that the adhesion between the coating and the microfoulant is low, probably due to the presence of a thin water lubrication layer. The latter observation is likely then, due to the microfoulant making direct contact with the hydrogel, and the shedding behavior is consistent with JKR adhesion theory, (Eq. 1). This indicates that we captured the correct contact mechanics, similar to PDMS 10:1, despite experiencing a hydrodynamic shear force that is an order of magnitude lower (fig. S7). We also see that for PEG-DA 10, practically all of the microfoulants are removed while for PDMS 10:1, a substantial fraction still remains.

**Fig. 4.**
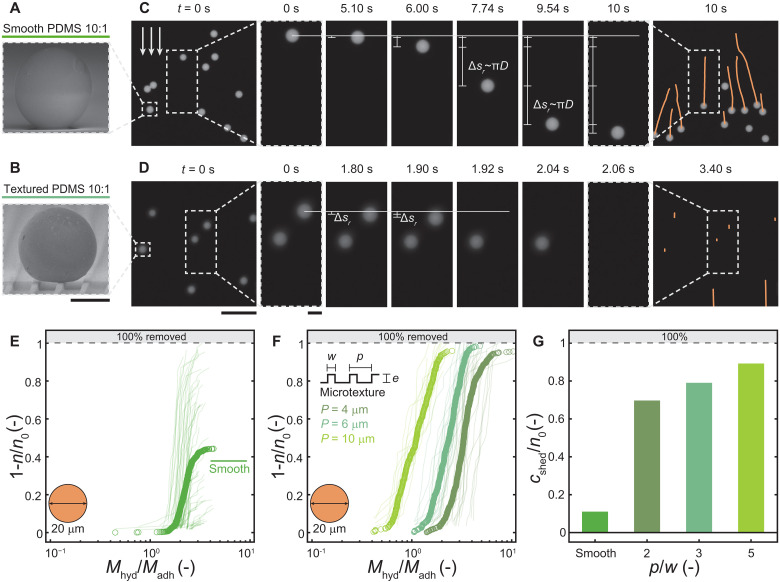
The effect of microfoulant size and microtexture on removal dynamics. Side-view micrographs showing the contact behavior of a microfoulant (*D* = 20 μm) on (**A**) smooth PDMS 10:1 and (**B**) microtextured PDMS 10:1 (δ ≈ 100 μm and surface texture, width *w* = 2 μm, height *e* = 2 μm, pitch *p* = 6 μm) coating in a vacuum environment. Epifluorescent image sequence showing the removal of microfoulants with *D* = 20 μm from a (**C**) smooth PDMS 10:1 and (**D**) microtextured PDMS 10:1 coating. Arrows display flow direction from top to bottom, microtexture aligned with the flow. Inset within sequence illustrates (C) representative rolling removal behavior indicated by the position markers and Δ*s_r_* matching the circumference length of the microfoulant and (D) shedding with initial small displacement on the coating. The last image shows the projected (*r*-ψ plane at *z* = 0) trajectories of the microfoulants in orange. (**E**) Removal efficiency (1 − *n*/*n*_0_) versus momentum ratio (*M*_hyd_/*M*_adh_) for smooth PDMS 10:1 and (**F**) microtextured PDMS 10:1 with varying *p*, keeping *w* and *e* constant. Circles represent the overall performance and semitransparent lines represent individual experiments, for *e* ≥ 14 experiments on *N* = 5 independent samples. (**G**) Occurrence of microfoulant shedding events *c*_shed_ from the coating, represented as *c*_shed_ /*n*_0_, versus smooth and microtextured coatings with varying *p*/*w*. Scale bars, (A) and (B) 10 μm; (C) and (D) first and last image, 200 μm; (C) and (D) inset image sequence, 20 μm.

To remove these additional microfoulants, our theoretical analysis reveals that it becomes crucial to modify *M*_adh_, which means minimizing *F*_adh_ and *a* of the compliant coating. One approach could be to reduce *W*_adh_, for example, by adding lubricants to the coating. The lubricants would cause a liquid layer and decreased stiffness, decreasing the performance, as we observed for PDMS 2:1 and CY52-276 coating (see Figs. 1H, 2, F and G, and 3C and figs. S4 and S5). An alternative approach is to reduce the contact area between the microfoulant and the coating by engineering the surface texture at a length scale smaller than the foulant. To see whether this approach works—and gain a more universal understanding of the removal behavior from our coatings across different microfoulant sizes—we choose to work with a microfoulant of diameter *D* = 20 μm. The variation in size is important because, for both particulate and crystallization fouling, the size of the foulants can vary. Furthermore, it enables us to study a wider range of diameter-to-surface feature size ratios similar to previous studies on microtextured antifouling coatings (*66*). Figure 4 (A and B) shows side-view micrographs of the microfoulant on smooth PDMS 10:1 and microtextured PDMS 10:1, respectively. Both coatings have a thickness of approximately δ ≈ 100 μm and the microtextured surface consists of ribs which have a width *w* = 2 μm, height *e* = 2 μm, and pitch *p* = 6 μm (see fig. S8 and table S1 for micrographs and roughness measurements of the smooth and microtextured surface).

Figure 4 (C and D) shows epifluorescent image sequences of smooth and rib microtextured PDMS 10:1 (see Materials and Methods) fouled with microfoulants (*D* = 20 μm) subjected to the same flow conditions as described in Fig. 2 for PDMS 10:1, with the flow direction being parallel to that of the ribs. The insets within the sequences display representative removal behavior of individual microfoulants along with the observed displacements, Δ*s_r_*. In addition, the last image displays the projected trajectories of the microfoulants in orange (*r*-ψ plane at *z* = 0). We observe for the *D* = 20 μm microfoulants on smooth PDMS 10:1 that the majority of microfoulants exhibit—in contrast to the shedding for the smaller size—a different mechanism: rolling motion (see movie S5). This rolling behavior on the compliant coating can be confirmed by examining small brighter spots on the microfoulant shell and combining it with the observed displacement measurements, which matches the circumference of the microfoulant, Δ*s_r_* ≈ π*D* (see movie S5). Figure 4 (E and F) shows plots of 1 − *n*/*n*_0_ versus *M*_hyd_/*M*_adh_ for the smooth and microtextured PDMS 10:1 coating, respectively. For the microtextured surfaces, we kept *w* and *e* constant while varying *p*, and we modified Eq. 1 to account for the microtexture (see fig. S9). From this, we can see that there is a mixture of rolling and shedding removal events, with the latter being more desirable, and so we also quantify the number of microfoulants that are removed, *c* = *n*_0_ − *n*, due to shedding, *c*_shed_ (Fig. 4G).

We see from this that the rolling motion leads to a substantial decrease in the removal performance, with a value of 1 − *n*/*n*_0_ = 0.44, compared to the smaller microfoulants in Fig. 3E, where the removal performance is 1 − *n*/*n*_0_ = 0.84. (For PEG-DA 10, we observe no performance decrease for the microfoulants of size *D* = 20 μm. See fig. S10 for representative removal image sequence.) This result highlights the counterintuitive behavior that we observe: For increasing *D*, we would expect the required hydrodynamic force for removal to decrease, leading to improved performance (*67*), while we observe the opposite. Furthermore, it highlights the importance of removing microfoulants or growing crystallites already at small sizes as the removal efficiency decreases with increasing size. For the microtextured PDMS 10:1, we observe that the microfoulants predominantly exhibit shedding behavior, with an initial small displacement Δ*s_r_*, similar to the experiments conducted with the smaller microfoulants on PDMS 10:1 (Fig. 2D; see movie S5 for removal dynamics). As anticipated, the microtexture promotes shedding which in turn increases the removal efficiency resulting in 1 − *n*/*n*_0_ > 0.95 for all microtextured coatings. We also observe that as *p* increases, for a given value of 1 – *n*/*n*_0_, *M*_hyd_/*M*_adh_ decreases, however only by a factor of roughly 5, showing that we still capture most of the removal mechanism, although we are clearly changing the nature of the contact and the flow conditions. We also observe a dependence of the hydrodynamic force necessary to remove a microfoulant on the orientation between the rib and the flow direction, with parallel orientation leading to the highest removal efficiency (fig. S11). For the variation of the ratio *p*/*w* (keeping *w* constant), we find an increased probability of shedding events with increasing ratio, which we attribute to the decrease in contact and increase of water flow beneath the microfoulant. However, we note that raising the pitch beyond a certain point (*p* − *w* ≈ *D*) would cause the microfoulants to get trapped in the rib microtexture, thereby increasing the contact, and negating any of the present benefits gained.

**Fig. 5.**
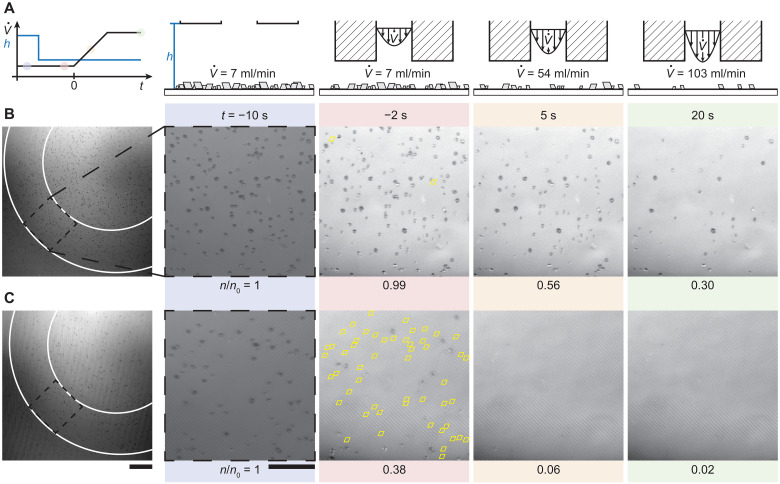
Rational design of compliant scalephobic coatings with intrinsic scale-shedding properties. (**A**) Schematic illustrates the volume flow $\dot{V}$ and gap height *h* versus *t*, colored points match the timestamps of the image sequences. Nozzle schematics indicate the nozzle position and the volume flow at specific times. Image sequence showing the removal of calcium carbonate crystallites from (**B**) smooth PEG-DA 50 and (**C**) microtextured (width *w* = 2 μm, height *e* = 2 μm, pitch *p* = 6 μm) PEG-DA 50 coatings. The first image in (B) and (C) indicates the reference state, the nozzle is far away (*h* = 2000 μm) from the surface, and white lines indicate the position of the nozzle for the image sequence. Yellow rhombus markers represent removed crystallites before the ramp-up of the volume flow. Scale bars, (B) and (C) first image, 200 μm; (B) and (C) image sequence, 100 μm.

Building upon the fundamental insights gained from studying model foulants, we seek to rationally design coatings with inherent scale-shedding properties. Figure 5A shows a schematic plot of the temporal evolution of $\dot{V}$ and *h*, indicating the nozzle approach to the surface at *t* = −7 s and the volume flow ramp-up starting at *t* = 0 s. The nozzle schematics show the nozzle position and the volume flow corresponding to the images in the sequence. Figure 5 (B and C) shows image sequences of a smooth PEG-DA 50 and a rib microtextured (*w* = 2 μm, *e* = 2 μm, *p* = 6 μm) PEG-DA 50, both having a polymer content of 50 wt %. The coatings have experienced crystallization fouling (see Materials and Methods) and are subjected to the same water shear flow conditions as the experiments presented in Fig. 1 (see fig. S2 for experimental volume flow data). The first magnified image inset shows both coatings at *t* = −7 s where the nozzle is far away (*h* = 2000 μm) from the surface and $\dot{V}$ = 7 ml min^−1^. The second image of the sequences shows the coatings at *t* = −2 s. We observe that for the smooth PEG-DA 50, most of the crystallites are still present *n*/*n*_0_(*t* = −2 s) = 0.99, while in the case of the microtextured PEG-DA 50, a substantial number of crystallites—yellow rhombus markers indicate removed crystallites—have already been removed *n*/*n*_0_(*t* = −2 s) = 0.38. At *t* = 20 s, we obtain *n*/*n*_0_(*t* = 20 s) = 0.30 for the smooth case and *n*/*n*_0_(*t* = 20 s) = 0.02 for the microtextured PEG-DA 50.

Previous research has shown that uniform, nonporous hydrogels with low swelling behavior require a polymer content of at least 40 wt % (*68*–*71*). Thus, to enhance the uniformity of the gel and avoid swelling-induced delamination or fracture of our coating, we opted to increase the polymer content of the PEG-DA coating to 50 wt %. In accordance with our understanding, we anticipate that the increase in polymer content negatively affects adhesion and removal. We showed in Fig. 1 (G and H) a decrease in performance, *n*/*n*_0_(*t* = 20 s) = 0.40 for PEG-DA 50 (movie S2) compared to *n*/*n*_0_(*t* = 20 s) = 0.11 for PEG-DA 10 (Fig. 1E), which we attribute to the decreased water content, meaning less stable lubrication and denser mesh (*69*) and therefore more contact between coating and scale. We can counteract this negative effect by taking the gained knowledge about surface texturing which improves removal performance and applying it to the more complex case of scale removal. The microtexturing of PEG-DA 50 with 50 wt % polymer content provides excellent removal performance where up to 98% of the crystallites are removed (see fig. S12 for more experiments). Furthermore, more than 60% of the crystallites are removed before we ramp up the volume flow, meaning removal at bulk flow velocities of $\bar{v}$ = 0.2 to 0.4 m s^−1^. This indicates the excellent design—scalephobicity—of our coating by combining high removal performance while reducing the necessary flow velocities and hydrodynamic forces for removal. We attribute the improved removal performance of PEG-DA coatings to an intervening water layer between the crystallites and the coating. The microtexture’s ability to further reduce the contact between the scale and the coating, especially for crystallites that are larger than *w*, promotes a stable water lubrication layer.

**Fig. 6.**
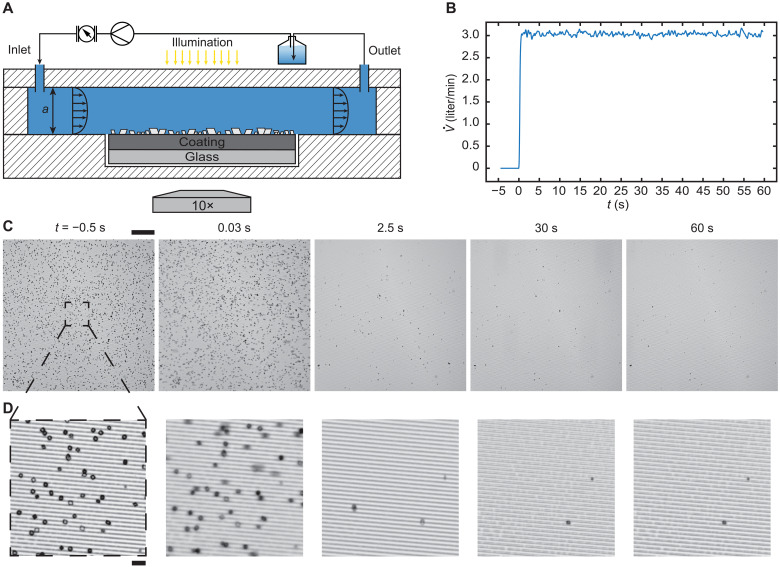
Shear-driven crystallite removal from microtextured PED-DA 50 in a parallel plate flow chamber. (**A**) Schematic (not to scale) of the test section. The polymethyl methacrylate chamber is composed of a parallel plate channel connected to a fluidic system (reservoir, pump and flowmeter), which provides a turbulent shear flow in the channel (height *a* = 3 mm, width *b* = 12 mm, length *l* = 120 mm, hydraulic diameter *D*_H_ = 4.8 mm; *Re* = ρ*uD*_H_/μ ≈ 6800; *u* ≈ 1.4 m s^−1^). (**B**) Experimental procedure showing $\dot{V}$, over time, *t.* Bottom-view image sequence showing the removal of calcium carbonate crystallites from (**C**) microtextured (width *w* = 2 μm, height *e* = 2 μm, pitch *p* = 6 μm) PEG-DA 50 coating. Flow direction left to right. (**D**) Magnified image sequence showing crystallite removal. Flow direction left to right. Scale bars, (C) 200 μm and (D) 20 μm.

To demonstrate the effectiveness of our designed scalephobic coating, we subject the coating to turbulent flow conditions as they would be present in heat exchangers (*72*, *73*). Figure 6A shows a schematic of the parallel plate flow setup that we used to test the removal behavior of our scalephobic coating (see Materials and Methods). We impose with a controlled pump a turbulent shear flow for 60 s (Fig. 6B) by pumping water through a parallel plate chamber with a hydraulic diameter, *D*_H_ = 4.8 mm. Figure 6C shows the bottom-view image sequence of microtextured (width *w* = 2 μm, height *e* = 2 μm, pitch *p* = 6 μm) PEG-DA 50 which has experienced crystallization fouling and is subjected to a turbulent water shear flow (*Re* = ρ*uD_H_*/μ ≈ 6800; *u* ≈ 1.4 m s^−1^). The magnified image sequence (Fig. 6D) shows that the microtexture aligns with the flow direction from left to right.

We find that the first crystallites get removed from the microtextured PEG-DA 50 almost immediately after initiating the flow (see movie S7). During that initial phase, a substantial number of crystallites are removed. In contrast, we do not observe this initial effect on the reference Glass substrate (fig. S13) although the samples are subjected to the same flow conditions. At *t* = 60 s, we obtain an almost clean surface demonstrating the scalephobicity of our designed coating under turbulent flow conditions.

## DISCUSSION

We have developed, based on adhesion and interfacial fluidic theories, a methodology to study the physics of microfoulant adhesion and removal in situ on submerged engineered compliant materials with the necessary spatial and temporal resolution. The development of the μ-sFDG methodology for analyzing antifouling materials can improve the approach to studying antifouling. The scanning approach provides local insight into the removal dynamics measurements with microscale spatial resolution, which is needed to understand the full behavior of the coating. We analyzed the behavior of more than >30,000 individual microfoulants of different types and sizes. Such an approach is complementary to a force-probe approach (*74*), focusing on frictional dynamics of repellent surfaces (*75*). By offering insights at the microscale, where fouling starts, and where one would aim to prevent it, this methodology can reveal how the foulants, substrate, and water interact and how removal occurs under flow conditions. We demonstrated that changing the wettability of rigid materials does not affect the removal performance, which is counterintuitive to previous literature (*20*, *36*) using ex situ methods. We revealed and quantified with a fundamental parameter space study that there are shedding, gliding, and rolling mechanisms governing the removal of microfoulants. Through microtexturing, we were able to alter the rolling mechanism and obtain the beneficial shedding which increased the removal performance while reducing the necessary hydrodynamic force. The presence of a water lubrication layer and the low adhesion lead to a gliding behavior of the foulants on the hydrogel.

On the basis of our findings, the design strategies for antifouling materials vary depending on the dominant fouling mechanism. When a surface experiences particulate fouling, rigid coatings perform well (fig. S1). When a surface experiences crystallization fouling, soft coatings outperform rigid coatings. The hydrogel, a compliant hydrophilic coating, with low polymer content showed excellent removal performance for both microfoulants and crystallites. For silicones, there is a possible trade-off of making the coating softer to enhance removal, but excessive compliance can lead to indentation (*76*) and lubrication-induced gliding that hinders removal. Introducing microtexture, as has been studied before for rigid materials (*8*, *36*), improved the performance of hydrophobic PDMS 10:1 for particulate fouling. However, in the case of crystallization fouling, we observed that microtexturing did not increase the performance compared to the smooth material (see fig. S14). Note that microtexturing of a more compliant material (e.g., *E* ≈ 10 kPa) is challenging due to the flattening of the texture caused by surface stresses (*48*). For the hydrophilic hydrogel, to be uniform and nonporous and to avoid delamination, the polymer content needs to be increased; however, a drawback of this approach is that the removal performance decreases, which can be mitigated by microtexturing the surface. We demonstrated coatings that had low adhesion to crystallization fouling deposits, one of the sought-after properties toward realizing intrinsically scalephobic surfaces (*17*). Specifically, we showed that by microtexturing soft hydrogel coatings, we can remove up to 98% of the crystallites, which is 66% better than rigid untreated substrate under the same shear flow conditions. We find similar behavior under turbulent flow conditions in a parallel flow condition, demonstrating scalephobicity in a more realistic environment. We anticipate that these findings can provide important information for the design of antifouling and scalephobic surfaces and can also be relevant for adhesion and interfacial transport research areas. Last, while our work focused on the removal of microfoulants, it opens up opportunities for further work on investigating the entire fouling process in situ under heat transfer and flow conditions, as would be present in many applications such as heat exchangers.

## MATERIALS AND METHODS

### Materials

Sylgard 184 (PDMS) and silicone CY52-276 (CY52-276) were purchased from Dow Corning. PEG-DA (*M*_w_ = 500 g mol^−1^), 2-hydroxy-2-methylpropiophenone (Darocur 1173), 3-(trimethoxysilyl)propyl methacrylate (TMSPMA), Rhodamine 6G, 1H,1H,2H,2H-Perfluorodecyltriethoxysilane (PFDTES), CaCl_2_, NaHCO_3_, CaCO_3_, and hexane were purchased from Sigma-Aldrich. Glass microscope slides (76 mm by 26 mm by 0.7 mm) were purchased from Marienfeld. Fluorescent polystyrene particles PS-FluoGreen (*D* = 10 μm, absorption/emission spectra: 502/518 nm) and SPHERO Yellow (*D* = 20 μm, absorption/emission: 470/490 nm) were purchased from Microparticles GmbH and Spherotech Inc. Macroscopic polystyrene particles (PSS-WHT-1.05 *D* = 6.3 mm) were purchased from Cospheric LLC.

### Coating preparation

All tested coatings were deposited onto glass substrates (22 mm by 22 mm by 0.7 mm). The substrates were cut to size using a diamond cutter. Before coating, the substrates were cleaned using bath sonication separately in acetone, then isopropyl alcohol, and lastly deionized (DI) water for 5 min each. Afterward, they were dried by blowing nitrogen gas on them.

#### PFDTES

We fabricated PFDTES-coated glass squares by chemical vapor deposition at high temperatures. To obtain a strong bonding between the silane and the glass, the squares were exposed to oxygen plasma for 5 min (Diener). Next, the squares were placed together with a vial of 80 μl of silane dissolved in 4 ml of hexane in an oven for 180 min at 95°C.

#### Preparation of mask for photolithography

We first designed the CAD drawing of the rib microtexture, width *w* = 2 μm, height *e* = 2 μm and varying pitches, *p* = 4, 6, and 10 μm, using open-source software K-layout. As a first step, we printed a mask using a laser writer (DWL 2000, Heidelberg Instruments). The laser directly writes the design on the photoresist. In this case, the substrate soda lime glass is coated with a thin layer of Cr (100 nm) and a positive photoresist (0.5 μm). The laser beam will expose the positive photoresist; the exposed areas will then be removed when the mask is developed. Afterward, the Cr layer from these areas will be etched in Cr etchant yielding a transparent/glass area everywhere we had a closed polygon in our design. After printing the mask, the photoresist was removed using a spray developer (AZ400 1:4) followed by drying with an N_2_ gun. Then, the Cr layer underneath was etched using a Cr etchant for 40 s, followed by rinsing the mask in DI water for 4 to 5 min and drying with nitrogen. Then, the remaining strip is removed using a TechniStrip (P1316) solution for about 10 to 15 min. Then, the mask was again rinsed in water and dried with nitrogen.

#### Silicon wafer fabrication

The silicon microtextured molds were fabricated through photolithography followed by deep reactive ion etching on a single-side polished silicon wafer at the cleanroom facilities of the Binnig and Rohrer Nanotechnology Center. We first treat the 4-inch silicon wafer inside a plasma cleaner for 3 min (600 W) to remove any organic contaminants. Then, the silicon wafer was pretreated with HMDS (hexamethyldisilazane) at 110°C for 1 min to promote the adhesion of photoresist on the inorganic wafer. A 1.2-μm-thick layer of AZ1512 photoresist was spin-coated at 4000 rpm for 40 s with a ramp of 2000 rpm s^−1^. The wafer was then baked at 110°C for 60 s. To transfer the pattern, the photoresist was exposed using a mask aligner (MA6) for 3.6 s. Then, the wafer was developed using an AZ developer (AZ400, 1:4). Next, we etched the wafer by deep reactive ion etching (Alcatel AMS 200SE I-Speeder) based on the etchant SF_6_ and C_4_F_8_ as the passivation layer. Post-etching, acetone was used to remove the positive photoresist from the silicon wafer followed by drying in nitrogen. As a last, step the wafer was treated with O_2_ plasma for 5 min (at 600 W) to remove any organic contaminants.

#### PDMS

We used polydimethylsiloxane, PDMS Sylgard 184, which we mixed in varying w/w ratios of 2:1, 10:1, 30:1, and 50:1 (pre-polymer to curing agent). The silicone elastomer was degassed for 10 min and then spin-coated (7000 rpm yielded δ ≈ 10 μm; 1000 rpm yielded δ ≈ 100 μm) onto the prepared glass substrates for 1 min. The samples were subsequently cured at 70°C for 60 min in an oven. Thicker samples (≈ 1000 μm) were fabricated using a laser-cut polymethyl methacrylate ring mold with the following dimensions: inner diameter, 15 mm; height, 1 mm. To fabricate the microtextured PDMS 10:1, we used the prepared silicon wafer as the mold. The PDMS 10:1 mixture was poured over the silicon negative and degassed again to remove all gas bubbles. After the curing step at 70°C for 60 min in an oven and storing for 2 weeks, the PDMS (δ ≈ 100 μm) was peeled off from the silicon mold, and the samples were cut to 22 mm–by–22 mm size and placed on the prepared glass substrates.

#### CY52-276

The two components of the silicone were mixed at a weight ratio of A:B of 5:6 for 3 min, degassed for 2 min, and spin-coated onto a microscope slide for 1 min (6000 rpm, δ ≈ 10 μm; 600 rpm, δ ≈100 μm). The silicone was then cured at 70°C for 30 min. The thicker samples (δ ≈ 1000 μm) were fabricated using an O-ring mold made of polymethyl methacrylate (see above). After preparation, samples were stored in a dry, dust-free environment to prevent contamination. To account for the aging properties of dimethyl siloxanes, we performed experiments on samples of similar age (2 to 3 weeks) (*77*).

#### PEG-DA

We prepared 5 g of 10 wt % (0.5 g) and 50 wt % (2.5 g) PEG-DA monomer base solution in DI water. We added 2 wt % (0.01 and 0.05 g, based on the added monomer amount) of the photoinitiator 2-hydroxy-2-methylpropiophenone (Darocur 1173) to the solution. To obtain a strong bond between the hydrogel and the glass squares, we exposed the squares to oxygen plasma for 5 min (Diener). Next, the glass squares were immersed in a 20-ml solution containing 0.3 ml of TMSPMA, 9.85 ml of ethanol, and 9.85 ml of DI water for 60 min. The squares were then washed with DI water and dried with nitrogen. Before ultraviolet (UV) cross-linking, 7 μl (δ ≈ 10 μm) and 70 μl (δ ≈ 100 μm) of the hydrogel solution were placed with a pipette on the pretreated glass squares and covered with an 18 mm–by–18 mm cleaned coverslip to spread the solution. The solution was cured with UV light for 5 min (15-W power and 350-nm wavelength). After the curing step, the coverslip was removed with a tweezer, and the samples were washed with DI water and left in a humid environment before testing. To fabricate the microtextured PEG-DA 50, we poured polyurethane acrylate (PUA) onto the patterned PDMS 10:1 positives, which we obtained from the silicon mold, and covered it with a TMSPMA-pretreated glass square. The material was cured with UV light for 5 min (15-W power and 350-nm wavelength). Next, PDMS 10:1 was peeled off and 70 μl of the 50 wt % PEG-DA solution was poured over the PUA mold and covered with a TMSPMA-pretreated glass square. After UV curing (15-W power and 350-nm wavelength) for 5 min, the PUA mold was separated under water from the PEG-DA substrate using a tweezer. The samples were washed with DI water and stored in a humid environment before testing.

### Material characterization

For the mechanical testing, we prepared macroscopic samples from the same mixtures that were used for coating the glass substrates. Approximately 5 g of PDMS 10:1, CY52-276, or PEG-DA 10 was cured in glass vials. The Young’s Modulus, *E*, of bulk materials was determined through mechanical testing using a texture analyzer (Stable Microsystems) equipped with a cylindrical indenter (2 mm). The force response during indentation was analyzed using MATLAB, accounting for the sample’s thickness (*78*). For the work of adhesion tests, we replaced the cylindrical indenter with a polystyrene sphere (diameter, 6.3 mm) and used a water environment by adding DI water to the vial. The maximum pull-off force was used to calculate the work of adhesion. For PEG-DA 10, we were not able to measure the work of adhesion, *W*_adh_, due to the low pull-off force, which was below the resolution limit of the texture analyzer. Therefore, we used a hydrogel literature value for PEG-DA (*62*) (*W*_adh_ = 0.001 J/m^2^). Contact angle measurements were performed on coated glass substrates with a DataPhysics OCA 35 goniometer using slow dosing rates to account for the rate-dependent wettability on compliant materials.

### Crystallization fouling protocol

For scale removal experiments, we prepared two solutions in DI water: (A) 30 to 40 mM CaCl_2_ and (B) 30 to 40 mM NaHCO_3_. To create the fouling solution with a supersaturation of 4.0 to 4.3 with respect to calcite (*79*), we mixed solutions A and B in a 1:1 ratio in a glass vial. We induced fouling by placing 600 μl of this solution onto the samples and stopped the growth of the crystals after 10 min by adding DI water to obtain crystals with a size of 5 to 15 μm. We performed all experiments within 10 min after adding DI water.

For the scale removal tests in the parallel plate chamber, we prepared the same solutions as mentioned above (volume, ~10 ml). We induced fouling by pumping the solution into the chamber. After 10 min, we performed the removal experiment.

### Particulate fouling protocol

For studying particulate fouling deposit removal (fig. S1), we prepared a dispersion of ground calcium carbonate powder in DI water followed by sonication. We placed 600 μl of the dispersion onto the mounted sample and let the particles settle for 10 min before we added DI water.

### Microfoulant fouling protocol

For model fouling (fluorescent polystyrene particles) removal experiments, we washed the polystyrene particles (10 and 20 μm) in water and stored them in buffer solution until usage. We placed 600 μl of 0.02 wt/vol % suspension onto the mounted sample in the experimental setup and allowed the particles to settle for 30 min before we added DI water. We performed all experiments within 15 min of adding DI water.

### Microscopic shear removal experiments using the μ-sFDG

With the goal of evaluating the effect of substrate compliance, composition, and texture on the in situ removal dynamics of crystallites and model foulants, we designed and developed a μ-sFDG inspired by previous work (*42*). A schematic of the full experimental setup is shown in fig. S6. By imposing a tunable laminar water shear flow close to a surface, we generate a shear stress which eventually leads to the removal of microfoulants which we observe through microscopic imaging. To impose the flow, we use either a controllable gear pump (Micropump, GAF T23) or a syringe pump (Harvard PHD ULTRA). These pumps operate in a wide range of flow rates, from 2 to 500 ml/min or 1 to 5000 μl/min. We connect the pump to a volume flowmeter (Endress+Hauser, Proline Cubemass C300) which accurately measures the volume flow (±0.1%). The pump and gauge system are in series connected to the inlet of a polished borosilicate glass capillary nozzle (length, 100 mm; inner radius, 560 μm; outer radius, 1000 μm). In the case of crystallite experiments, we coated the shell of the capillary (nozzle) with a 1:100 mixture of Rhodamine 6G and glue (merz+benteli ag, Cementit). We excite the Rhodamine 6G to obtain a back illumination from the nozzle shell to visualize the crystallites. We mounted the nozzle on a kinematic rotation stage (KKD 25C, OptoSigma) and a linear piezo stage (SLC 1730 SmartAct GmbH) (±1 nm). The stages serve two purposes: first, to ensure that the nozzle is aligned parallel to the tested substrate, and second, to regulate the height (*h* = 80 μm) of the resulting gap between the nozzle and the substrate precisely. Up to six samples are clamped in a two-piece home-built holder which ensures optical access from the bottom and water tightness using an O-ring. A connected peristaltic pump maintains a constant water level in the holder and transports the fluid back into the main reservoir where the temperature is monitored with a K-type thermocouple. To obtain the scanning function of the setup, we mounted the holder on the nano-piezo stage (*z* alignment) and micro stage (*x*-*y*-*z* movement) of an inverted fluorescent microscope (Nikon, Eclipse Ti2-E) which allows the home-built software to do automated removal experiments at up to 10 spots on a sample. The removal was captured through a 10× objective (Nikon Plan Apo Lambda D 10×) at 50 fps with a scientific complementary metal-oxide semiconductor (CMOS) camera (HAMAMATSU ORCA-Flash4.0 V3). All sensor control, data acquisition, and triggering were obtained and synchronized with the Nikon microscope using a data acquisition system (Beckhoff) and LabView.

With this setup, we can carry out removal experiments for microfoulants: Once the fouling process is complete, and the water level in the sample holder is stabilized, we center the pre-aligned nozzle to the sample and bring it into the vicinity of the substrate (*h* = 80 μm). We initiate the scanning function, and the *x*-*y* stages move to 1 of 10 locations on the sample and automatically readjust the channel height *h* using the perfect focus system of the microscope to account for small deviations (≈ 5 μm) of the glass squares. To prevent the removal of the foulants, the nozzle is maintained at *h* = 2000 μm during the movement, while flow rates are kept at approximately 5% of the maximum flow rate. We never shut off the flow to avoid pressure drops or backflow. At the experiment location, the system captures a reference image and after stabilization of the flow and nozzle, the flow rate is ramped up over 10 s. In the case of crystallites, we keep the flow rate at the highest level for an additional 10 s. After the removal experiment, the system takes post-images for control and moves to the next location. In the case of patterned samples and crystallites, the perfect focus system is ineffective because of the obstruction of light and low signal. Therefore, we manually focus on each location.

### Parallel plate chamber setup

The parallel plate chamber setup consists of a membrane pump (Shurflo 8000-543-290), which we connect to a flowmeter (Endress+Hauser, Proline Cubemass C300; accuracy: ±0.1%). The pump and flowmeter are connected in series before the inlet of a home-built polymethyl methacrylate chamber. To avoid flow disturbances in the test section, we mount the sample such that it is recessed into the chamber. We connect the outlet of the chamber to a reservoir to obtain a closed loop. We observe and capture the removal with an inverted bright-field microscope (Nikon, Eclipse Ti2-E) through a 10× objective (Nikon Plan Apo Lambda D 10×) at 30 fps with a scientific CMOS camera (HAMAMATSU ORCA-Flash4.0 V3). All sensor control, data acquisition, and triggering were obtained and synchronized with the Nikon microscope using a data acquisition system (Beckhoff) and LabView.

### Image processing and data processing

#### Microfoulants

All videos of the removal are processed using TrackMate (*80*) in Fiji with consistent tracking parameters to determine the spatial position of each foulant during removal. Only foulants below the nozzle are considered for data evaluation. Using an in-house MATLAB code, we determine the precise position of the nozzle (within ±5 μm) from a reference image taken before the ramp-up phase. The tracking data are combined with the sensor data to calculate the removal velocity and the forces acting on each foulant.

#### Crystallites

Figure S3 shows an image sequence of the image postprocessing. We first identify the nozzle position using an in-house MATLAB code to provide the region where crystallites are counted. We further process the image using background subtraction, binarization, and nonlocal means denoising in Fiji ImageJ. We detect the crystallites using in-house MATLAB code and obtain the number of crystallites *n* for each image.

## Supplementary Materials

### This PDF file includes:

Figs. S1 to S14 Table S1 Note S1 Legends for movies S1 to S7 References
