# Supplementary material for: Imparting scalephobicity with rational microtexturing of soft materials
Source: Sci Adv. 2023 Dec 20;9(51):eadj0324. doi: 10.1126/sciadv.adj0324 (PMC10732533; doi:10.1126/sciadv.adj0324)
Supplement: Supplementary file 1 — Figs. S1 to S14 Table S1 Note S1 Legends for movies S1 to S7 References [file sciadv.adj0324_sm.pdf]

Science Advances
AAAS

Supplementary Materials for

Imparting scalephobicity with rational microtexturing of soft materials

Julian Schmid *et al.*

Corresponding author: Thomas M. Schutzius, tschutzius@berkeley.edu

Sci. Adv. **9**, eadj0324 (2023)
DOI: 10.1126/sciadv.adj0324

The PDF file includes:

Figs. S1 to S14
Table S1
Note S1
Legends for movies S1 to S7
References

Other Supplementary Material for this manuscript includes the following:

Movies S1 to S7

Supplementary Materials

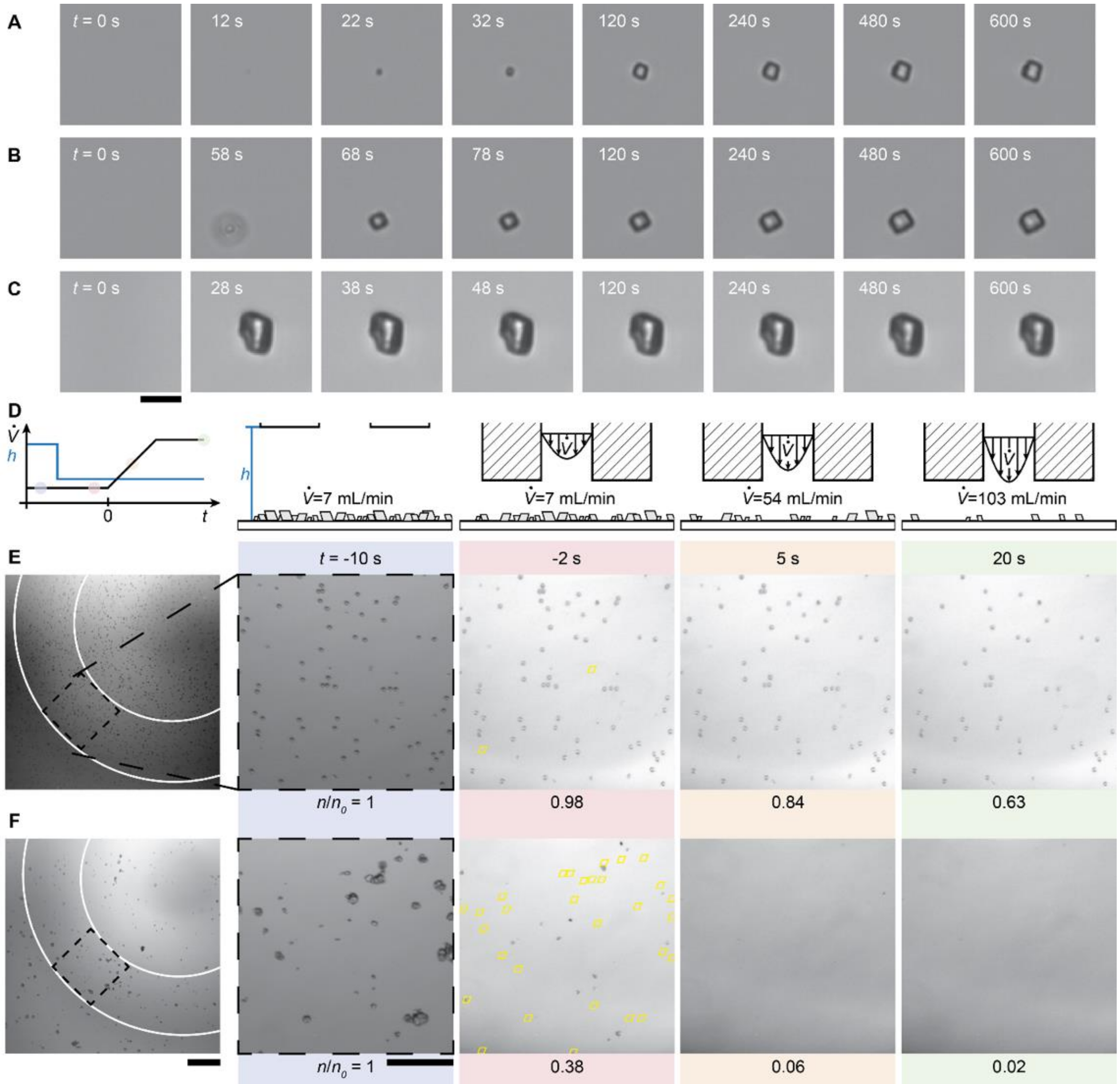

Figure S1**. Calcium carbonate crystallization fouling vs. particulate fouling.** (**A**) Brightfield bottom-view image sequence showing a crystallite nucleating and growing on a glass substrate from an aqueous supersaturated calcium chloride-sodium bicarbonate solution (see Materials and Methods). (**B**) Microscopic crystallite settling and growing on a glass substrate from an aqueous supersaturated calcium chloride-sodium bicarbonate solution (see Materials and Methods). In both cases, the crystallites grow on the substrate producing scale deposits until we add deionized water, interrupting further growth (*t* > 600 s). (**C**) Image sequence showing a calcium carbonate particle settling on a glass substrate from a calcium carbonate particle-water dispersion, producing a particulate deposit. This dispersion was made by combining calcium carbonate powder with deionized water and sonicating (Materials and Methods). Here we do not observe any crystallite growth on the substrate. We measured the difference in adhesive behavior between the crystallites that settled on the surface compared to those that grew on the surface, using the μ-sFDG setup.

(**D**) Volume flow $\dot{V}$ and gap height h vs. time, t. Image sequence showing the adhesive behavior of (**E**) scale deposits vs. (**F**) particulate deposits on glass substrates. The first image in (**E**), (**F**) indicates the reference state when the nozzle is far away (h = 2000 µm) from the glass-water interface, and the white lines indicate the inner and outer diameter of the µ-sFDG nozzle. Scale bars: (**A**)-(**C**) 10 µm; (**E**), (**F**) first image 200 µm; (**E**), (**F**) 100 µm.

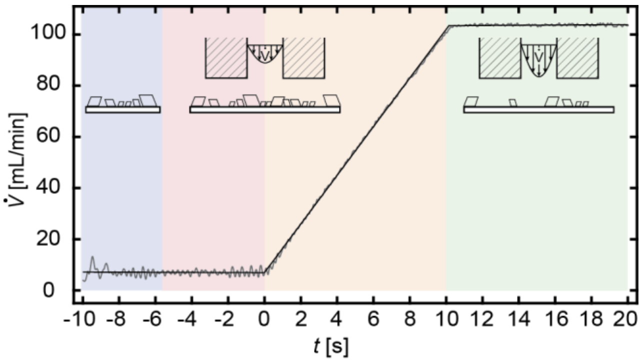

Figure S2. **Volume flow vs. time for crystallites removal experiments.** Gray line represents the mean volume flow over time of 5 different experiments, black line is the fitted line. The region with the blue background represents the period from t = -10 s to -7 s, during which the channel gap is *h* = 2000 µm. In the subsequent period from t = -7 s to 0 s, indicated by the red background, the nozzle is brought close to the surface *h* = 80 µm. From t = 0 s to 10 s, denoted by the orange background, the volume flow ramps up. The green background represents the constant volume flow region between *t* = 10 s and 20 s.

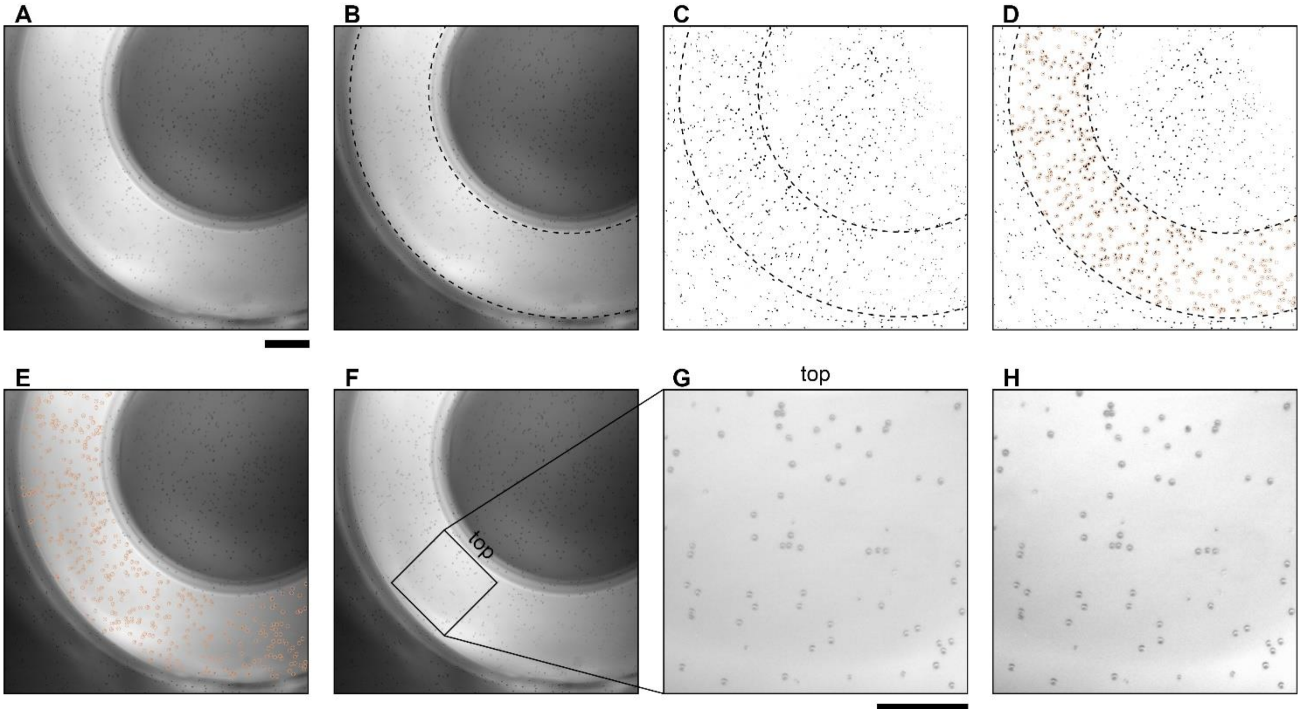

Figure S3. **Image postprocessing to obtain number of crystallites *n* on the surface and processing for image sequences.** The image in (**A**) shows an unprocessed raw image. (**B**) nozzle detection using in-house MATLAB code to provide region where crystallites are counted (**C**) processed image after background subtraction, binarization, and non-local means denoising using ImageJ with overlayed nozzle position (**D**) object detection using in-house MATLAB code, orange circle represent detected crystallites. We obtain the number of crystallites *n* by counting the individual detected objects. (**E**) overlay of detected crystals with the raw image showing excellent detection quality. (**F**) Raw image with box to indicate the region which we crop for the image sequences in Figure 1. (**G**) Cropped raw image, rotated to obtain flow from top to bottom. (**H**) image after linear adjustments of brightness. Scale bars: (**A**)-(**F**) 200 µm; (**G**)-(**H**) 100 µm.

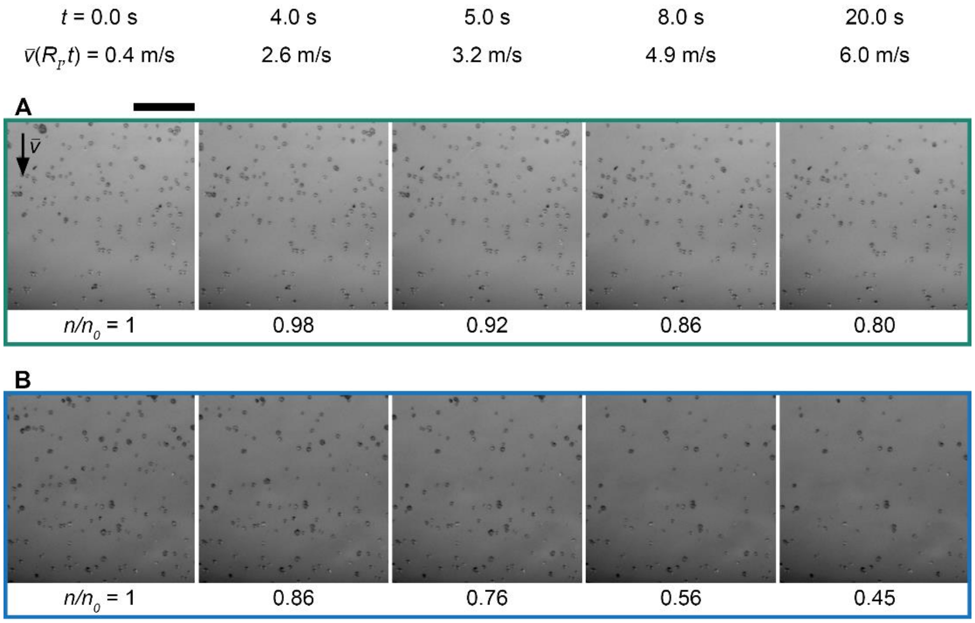

Figure S4. **Water shear-driven microfoulant dynamics on compliant coated substrates.** Bottom-view image sequence showing calcium carbonate crystallites on (**A**) CY52-276 and (**B**) PEG-DA 50 coated glass immersed in water and subjected to a shear flow (starting at $t = 0$ s, the flow rate increases from 7 to 103 mL/min in a channel of 80 µm height). Scale bar: 100 µm.

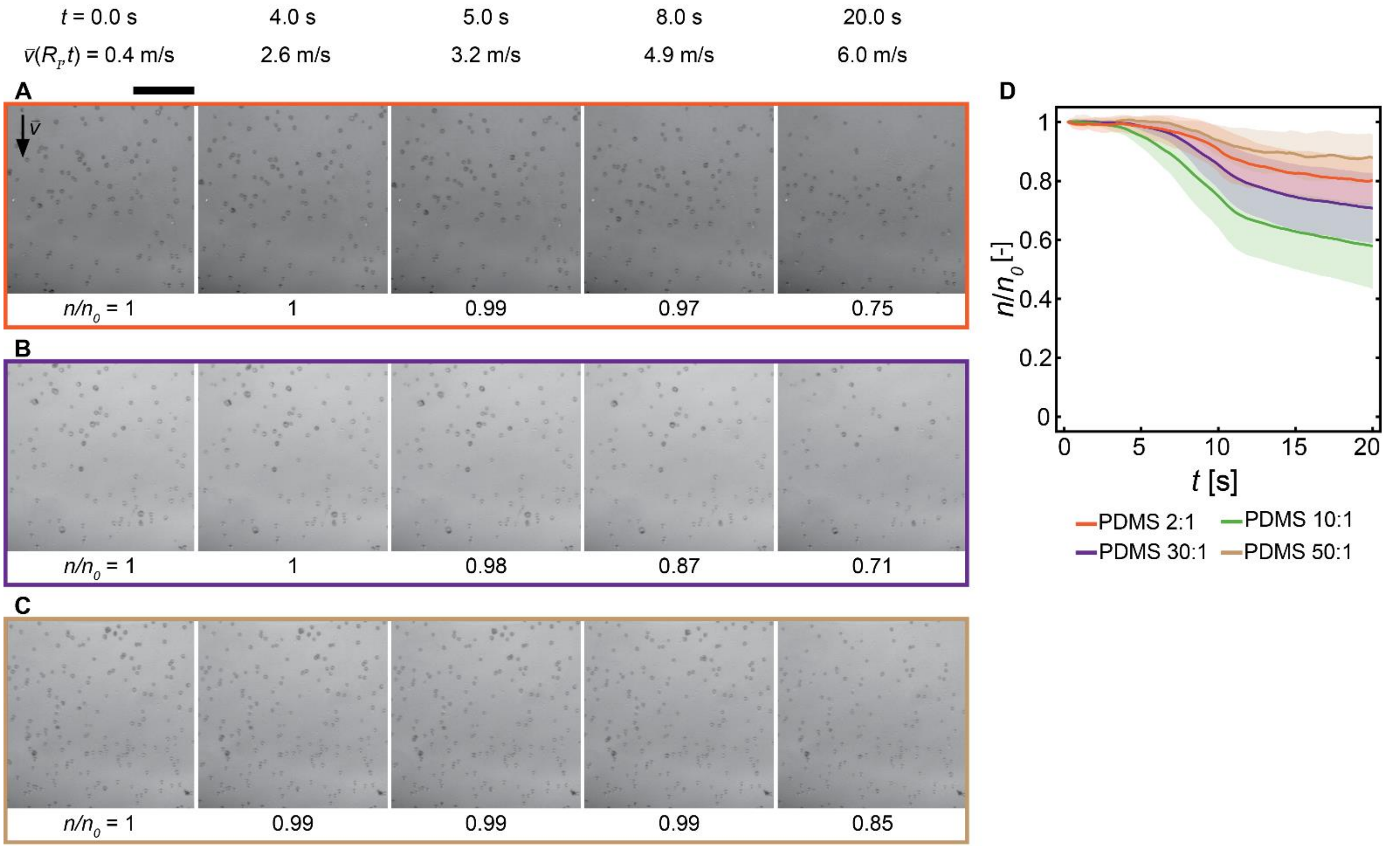

Figure S5. **Microfoulant dynamics under shear-driven water flow on PDMS coated substrates**: Bottom-view image sequence showing calcium carbonate crystallites on (**A**) PDMS 2:1 coated glass, (**B**) PDMS 30:1 coated glass and (**C**) PDMS 50:1 coated glass (coating thickness $\delta \approx 10$ µm) immersed in water and subjected to a shear flow (starting at $t = 0$ s, the flow rate increases from 7 to 103 mL/min in a channel of 80 µm height, resulting in a bulk velocity, $\bar{v} = 0.2$ m s^{-1} to 6 m s^{-1}). (**D**) Temporal evolution of n/n_0 for the various PDMS coatings on glass substrates. Lines representing the mean values and shaded regions are the standard deviation for $e \geq 9$ experiments on $N = 3$ independent samples. Scale bars: (**A**)-(**C**) 100 µm.

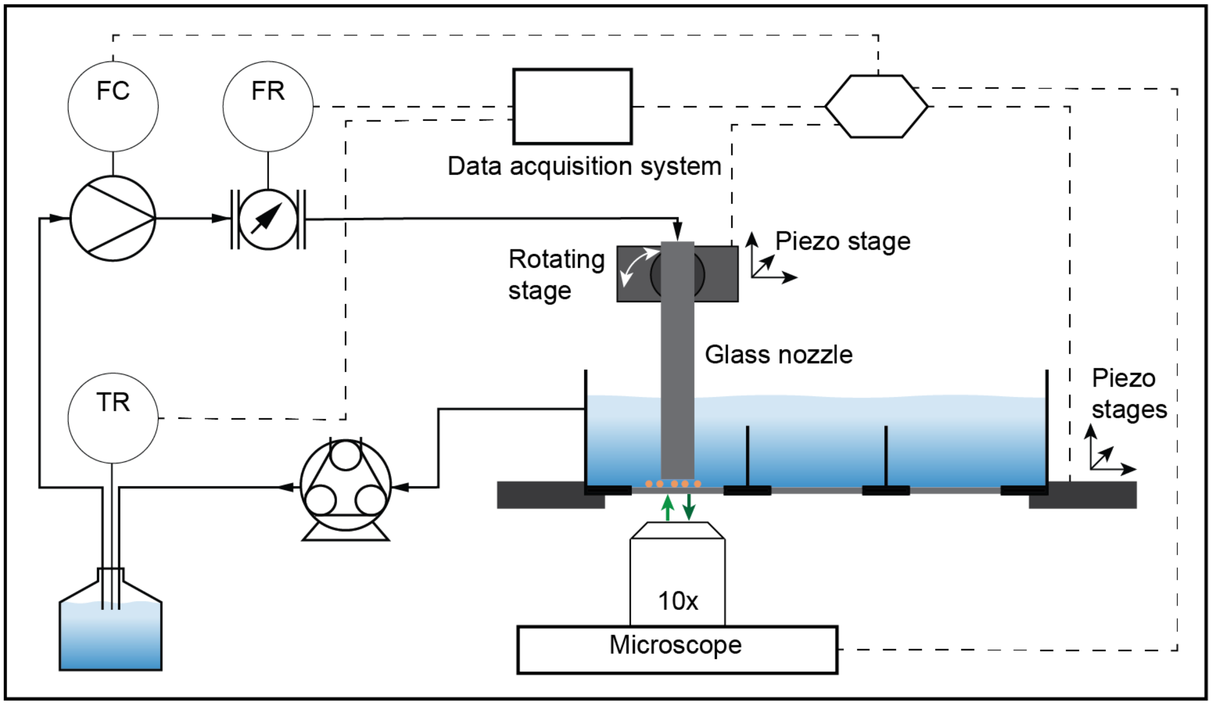

Figure S6. **Schematic of the µ-sFDG setup**. Schematics showing the developed and designed micro scanning fluid dynamic gauge system (µ-sFDG) inspired by previous work (*42*, *82*, *83*). The controllable pump, pump the water from the reservoir through a volume flow gauge system to the inlet of a glass capillary nozzle. The piezo stages allow the nanometer scale alignment of the nozzle parallel to the surface of the tested coating. The home-built holder, mounted on nano-micro piezo stages of a fluorescent inverted microscope (10x magnification, 50 FPS) can take up to six transparent samples. The peristaltic pump maintains a constant water level in the holder and pumps back the fluid to the temperature monitored reservoir. The data acquisition system controls and synchronizes all sensors, data acquisition, and triggering coupled to the Nikon microscope (see Materials and Methods for details on the used devices).

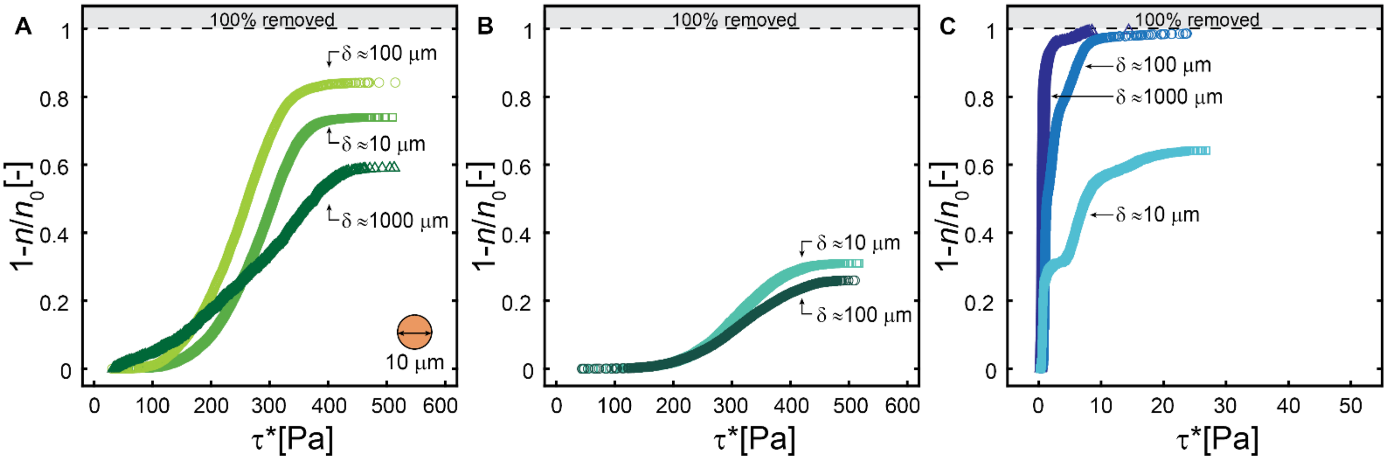

Figure S7. **Influence of coating thickness on microfoulant removal.** By knowing the flow condition in the channel and by observing the moment of foulant detachment the critical shear stress acting on the foulant can be approximated based on a radial laminar Poiseuille flow (*83*)as $\tau^* \approx 3\mu\dot{V}/(4\pi(h/2-D)^2 s_r)$, where μ is the dynamic viscosity of the liquid, *h* the channel gap height, *D* the particle diameter, s_r the microfoulant position below the nozzle and $\dot{V}$ the flow rate at the time of removal. Removal efficiency 1-n/n_0 vs, critical shear stress τ^* acting on the microfoulant for varying coating thicknesses, square symbol δ ≈ 10 μm, circle δ ≈ 100 μm and triangle δ ≈ 1000 μm for (**A**) PDMS 10:1, (**B**) CY52-276 and (**C**) PEG-DA 10. For CY52-276 no removal occurred for δ ≈ 1000 μm.

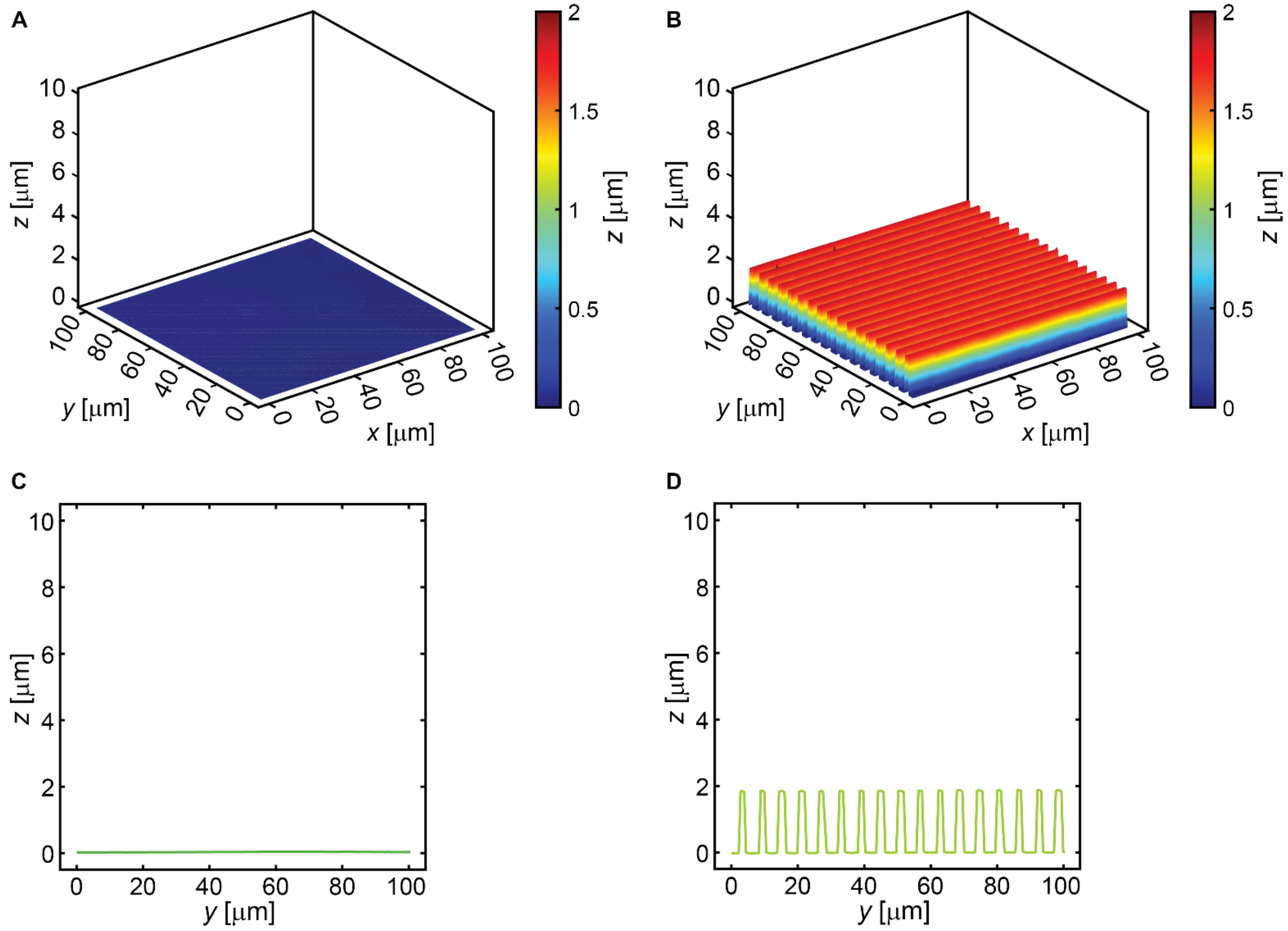

Figure S8. **Microscale roughness analysis of soft materials**. White light interferometry (See Materials & Methods on details of the measurements) 3D micrograph of glass coated with (**A**) PDMS 10:1, RMS roughness 3.5 nm and (**B**) microtextured PDMS 10:1, RMS roughness 861.6 nm. (**C**) and (**D**) show extracted 2D line topography measurements for the samples shown in (**A)** and (**B)** at x=50μm.

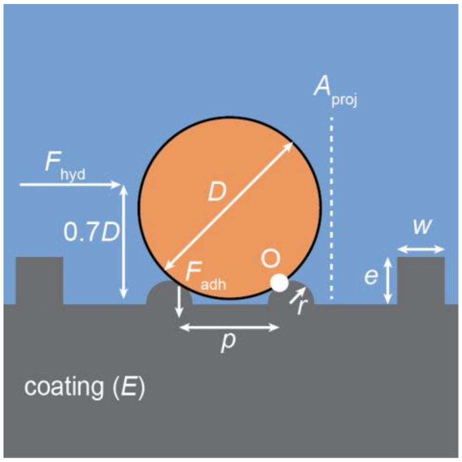

Figure S9. **Modification of moment analysis to account for surface microtexture**. Schematics show the modified theoretical analysis to account for the surface microtexture. From our SEM images we obtain that the particle tends to take a stable position between the texture ribs. Similar to previous research (*61*), we assume for $r \ll D/2$ that the contact between the particle and the texture can be described by a contact of a small particle of radius r with a flat surface, in this case the particle. Also, the influence of the texture height e is negligible if the particle is considerably larger than the texture. For our anisotropic texture, the particle can either be removed along the rib texture or perpendicular. We model the latter case to account for the fact that only a small portion of the particles measured in our experimental design undergo removal aligned with the texture. The hydrodynamic moment can be described as $M_{\mathrm{hyd}} = 0.7D\, F_{\mathrm{hyd}}\sqrt{(D/2)^2 - (p/2)^2}$ and the adhesion moment as $M_{\mathrm{adh}} = 3\pi W_{\mathrm{adh}} r\, p$, which is a product of F_{adh} at one rib and the distance p between the ribs.

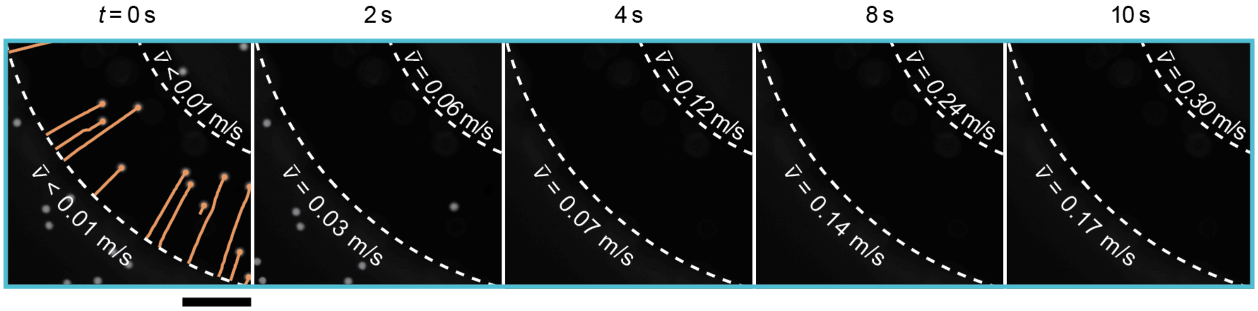

Figure S10. **Shear removal of microfoulants with *D* = 20 µm from PEG-DA 10 coating**. Epifluorescent bottom view image sequence showing the position of the microfoulants beneath the nozzle (dashed circular lines) and the bulk flow velocities $\bar{v}$, imparted from the water flow to the surface at $r = R_I$ and R_O, respectively, on PEG-DA 10 ($\delta \approx 100$ µm). The image at time-zero shows the projected (r-ψ plane at $z = 0$) trajectories of removed microfoulants in orange. Scale bar: 200 µm.

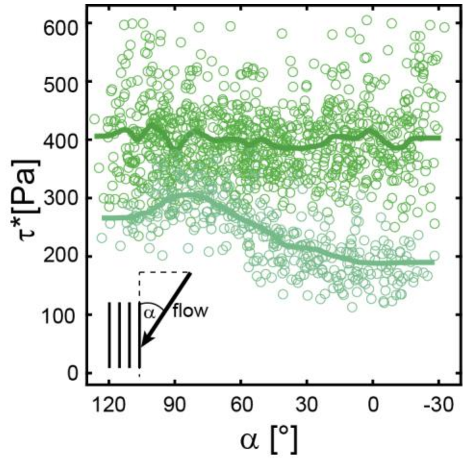

Figure S11. **Influence of microtexture and flow orientation on critical removal shear stress.** Critical removal shear stress τ^* versus flow angle of attack α for green smooth PDMS 10:1 and mint green microtextured (width *w* = 2 µm, height *e* = 2 µm, pitch *p* = 6 µm) PDMS 10:1. Lines represent moving mean value. The angle α is defined to be 90° if the flow is perpendicular to the orientation of the rib microtexture and 0° if the flow aligns with the microtexture.

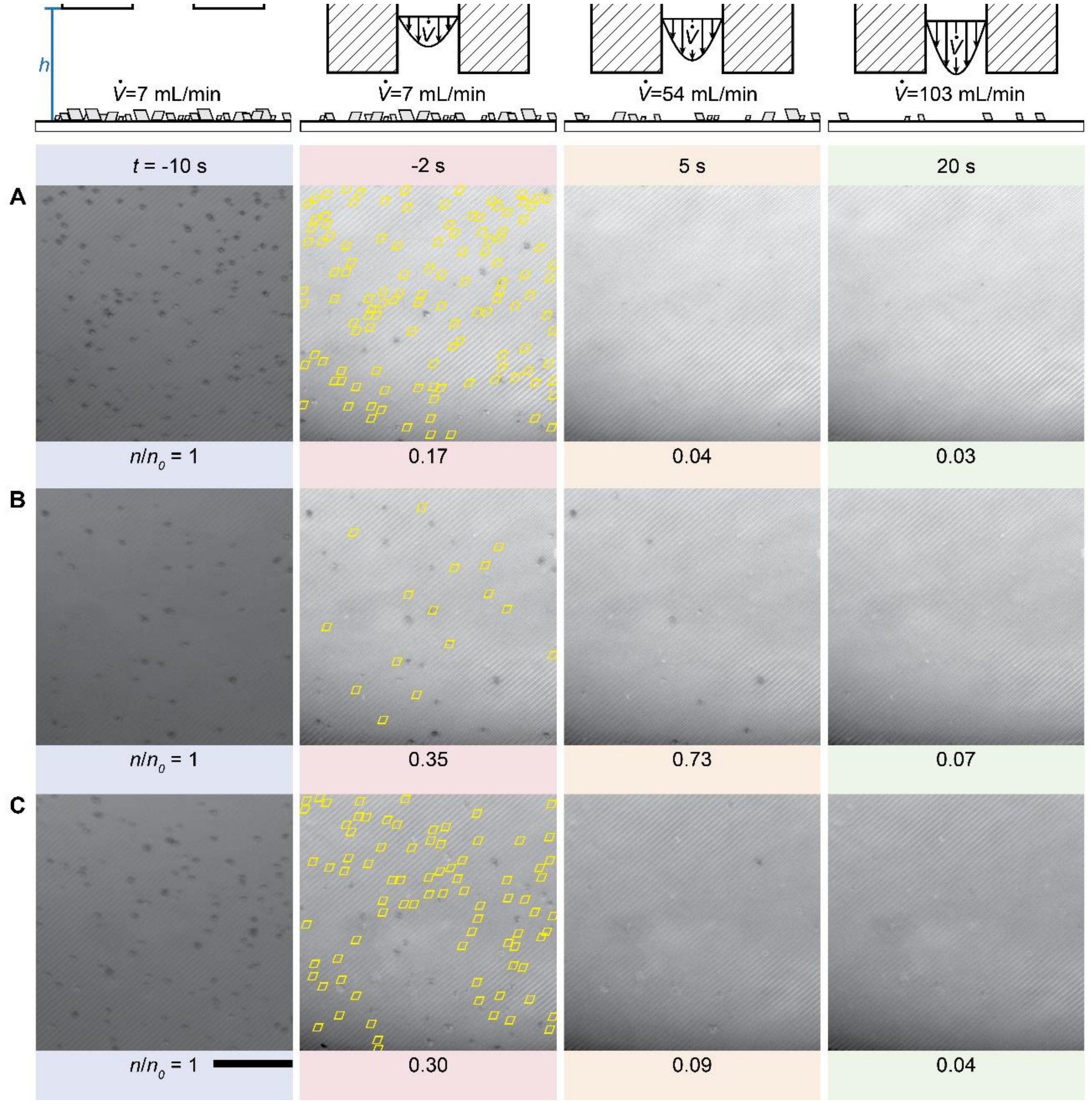

Figure S12. **Additional image sequences of crystallite removal from microtextured PEG-DA 50.** Image sequence showing the removal of crystallites from microtextured (width w = 2 µm, height e = 2 µm, pitch p = 6 µm) PEG-DA 50 for three independent samples (**A**)-(**C**). Nozzle schematics indicate the nozzle position and the volume flow at specific times. Yellow rhombus markers represent already removed crystallites before the ramp up of the volume flow. Scale bars: (**A**)-(**C**) image sequence 100 µm.

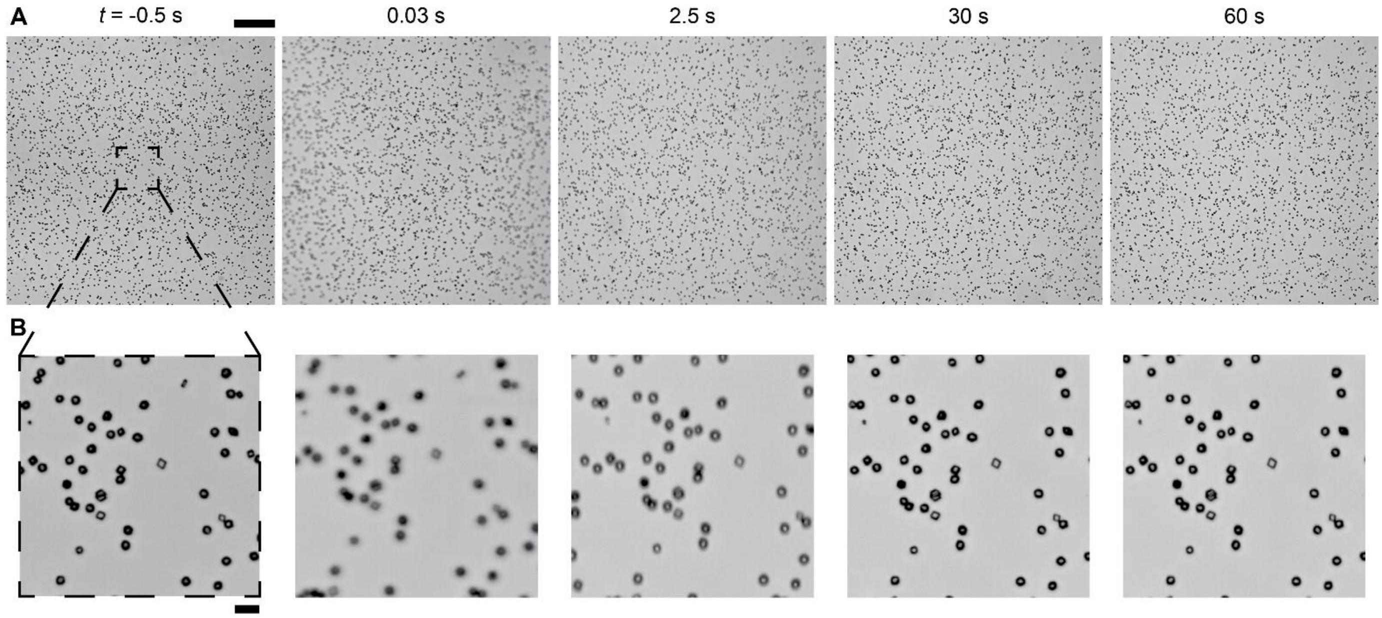

Figure S13. **Shear-driven crystallite removal from Glass in a parallel plate flow chamber**. (**A**) Bottom view image sequence showing the removal of calcium carbonate crystallites from cleaned Glass by imposing a turbulent shear flow ($Re = \rho u D_H/\mu \approx 6800$; $u \approx 1.4$ m s^{-1}). (**B**) Magnified image sequence showing that most of the crystallites are not removed. Flow direction left to right. Scale bars: (**A**) 200 µm; (**B**) 20 µm.

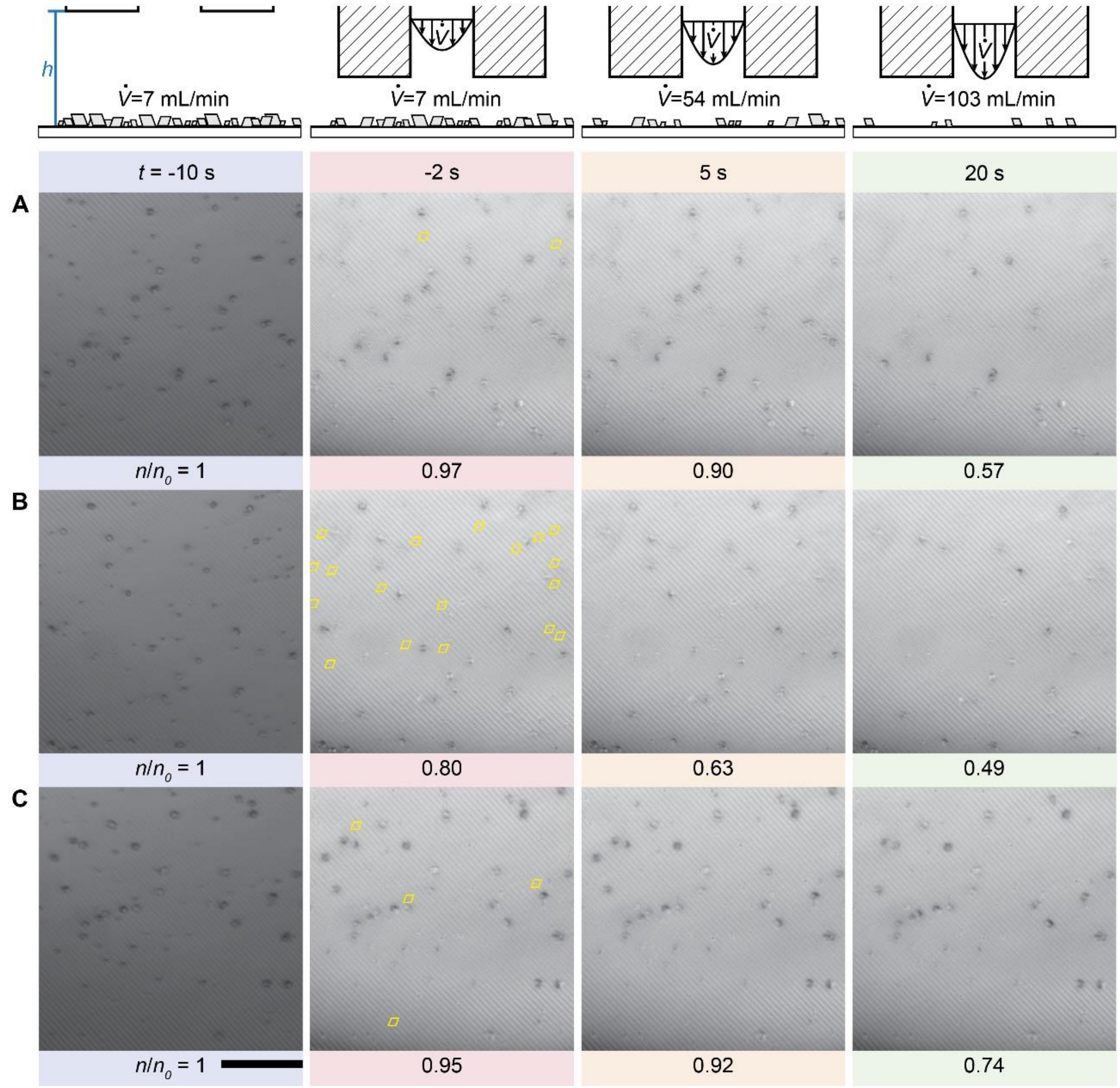

Figure S14. **Crystallite removal from microtextured PDMS 10:1.** Image sequence showing the removal of crystallites from microtextured (width w = 2 µm, height e = 2 µm, pitch p = 6 µm) PDMS 10:1 for three independent samples (**A**)-(**C**). Nozzle schematics indicate the nozzle position and the volume flow at specific times. Yellow rhombus markers represent already removed crystallites before the ramp up of the volume flow. Scale bars: (**A**)-(**C**) image sequence 100 µm.

Supplementary Information Tables

Table S1. **Surface Roughness measurements of smooth coatings and substrates**. Details on the measurements are provided in Materials and Methods.

**Smooth coatings**	**RMS (Sq) roughness [nm]**
CY52-276	1.4 ± 0.4
PDMS 10:1	6.5 ± 1.0
PEG-DA 10	3.5 ± 1.4

Supplementary Information Notes

Supplementary Note 1

Determining the drag coefficient C_D: The drag coefficient of a spherical particle in the bulk of a fluid can be expressed as $C_D = 24/Re_D$, where Re_D represents the particle Reynolds number given by $Re_D = \rho \bar{u} D / \mu$. In the case of a particle, or in our case a spherical foulant, which is attached to a surface a correction factor $f = 1.7009$ can be applied, to obtain $C_D = f\, 24/Re_D$ (*65*). It is important to note that this equation is only applicable when $Re_D \ll 1$. In our study, we need to account for higher Re_D especially for silicone coatings. Therefore, we use the following extensions (*84*):

$$C_D = \begin{cases} 1.7009 \dfrac{24}{Re_D}(1 + 0.0916 Re_D) & \text{for } 0.1 \leq Re_D < 5 \\ 1.7009 \dfrac{24}{Re_D}\left(1 + 0.158 Re_D^{2/3}\right) & \text{for } 5 \leq Re_D < 250 \end{cases}$$

Determining the mean flow velocity near the foulant $\bar{u}$: The velocity profile in the channel can be described by radial laminar Poiseuille flow, for $Re_{gap} = 2\rho \dot{V} / [\mu(2\pi R_I + h)] < 1400$ and a channel gap h, that is substantially less than the nozzle wall thickness, $R_o - R_I$. By knowing the radial position s_r at the moment of detachment the velocity profile near the foulant can be described as (*42*):

$$u(r, z, t) = \frac{3\dot{V}}{4\pi h} \frac{1}{s_r} \left[1 - \left(\frac{z - h/2}{h/2}\right)^2\right]$$

Therefore, we can determine $\bar{u}$ for $z \in (0, D - d)$ across the foulant.

Supplementary Information Videos

Video S1. Shear-driven removal of crystallites from compliant and rigid substrates that are hydrophilic.

Video S2. Shear-driven removal of crystallites from compliant and rigid substrates that are hydrophobic.

Video S3. Shear-driven removal of crystallites from PDMS with varying compliance.

Video S4. Shear-driven removal of microfoulants from compliant substrates.

Video S5. Microtexturing enhances shear-driven removal of microfoulants.

Video S6. Rationally designed compliant scalephobic coating with intrinsic scale shedding properties.

Video S7. Shear-driven crystallite removal in a parallel plate flow chamber.

REFERENCES

1. C. J. Vörösmarty, P. Green, J. Salisbury, R. B. Lammers, Global water resources: Vulnerability from climate change and population growth. *Science* **289**, 284–288 (2000).

2. United Nations, *Transforming Our World: The 2030 Agenda for Sustainable Development* (United Nations, 2015); https://sdgs.un.org/publications/transforming-our-world-2030-agenda-sustainable-development-17981.

3. U.S. Department of Energy, *The Water-Energy Nexus: Challenges and Opportunities* (Department of Energy, 2014); www.energy.gov/articles/water-energy-nexus-challenges-and-opportunities.

4. D. Magagna, I. Hidalgo González, G. Bidoglio, S. Peteves, M. Adamovic, B. Bisselink, M. Felice, A. De Roo, C. Dorati, D. Ganora, H. Medarac, A. Pistocchi, *Water – Energy Nexus in Europe*" (Luxembourg: Publications Office of the European Union, 2019).

5. J. He, T. M. Tritt, Advances in thermoelectric materials research: Looking back and moving forward. *Science* **357**, eaak9997 (2017).

6. R. Ghosh, T. K. Ray, R. Ganguly, Cooling tower fog harvesting in power plants—A pilot study. *Energy* **89**, 1018–1028 (2015).

7. M. Damak, K. K. Varanasi, Electrostatically driven fog collection using space charge injection. *Sci. Adv.* **4**, eaao5323 (2018).

8. J. Zhao, M. Wang, H. M. S. Lababidi, H. Al-Adwani, K. K. Gleason, A review of heterogeneous nucleation of calcium carbonate and control strategies for scale formation in multi-stage flash (MSF) desalination plants. *Desalination* **442**, 75–88 (2018).

9. J. J. Urban, Emerging scientific and engineering opportunities within the water-energy nexus. *Joule.* **1**, 665–688 (2017).

10. M. R. Malayeri, A. Al-Janabi, H. Müller-Steinhagen, Application of nano-modified surfaces for fouling mitigation. *Int. J. Energy Res.* **33**, 1101–1113 (2009).

11. M. Chaussemier, E. Pourmohtasham, D. Gelus, N. Pécoul, H. Perrot, J. Lédion, H. Cheap-Charpentier, O. Horner, State of art of natural inhibitors of calcium carbonate scaling. a review article. *Desalination* **356**, 47–55 (2015).

12. M. S. Abd-Elhady, M. R. Malayeri, Fouling mitigation in tubular exchangers using various projectiles: Deposit removal mechanisms. *Chem. Eng. Technol.* **38**, 2226–2234 (2015).

13. K. L. Petersen, A. Paytan, E. Rahav, O. Levy, J. Silverman, O. Barzel, D. Potts, E. Bar-Zeev, Impact of brine and antiscalants on reef-building corals in the Gulf of Aqaba—Potential effects from desalination plants. *Water Res.* **144**, 183–191 (2018).

14. M. Crabtree, D. Eslinger, P. Fletcher, M. Miller, A. Johnson, G. King, Fighting scale: Removal and prevention. *Oilfield Rev.* **11**, 30–45 (1999).

15. R. Steinhagen, H. Müller-Steinhagen, K. Maani, Problems and costs due to heat exchanger fouling in new zealand industries. *Heat Transf. Eng.* **14**, 19–30 (1993).

16. Z. Yan, D. Zhou, Q. Zhang, Y. Zhu, Z. Wu, A critical review on fouling influence factors and antifouling coatings for heat exchangers of high-salt industrial wastewater. *Desalination* **553**, 116504 (2023).

17. Y. Wang, J. Meng, S. Wang, Recent progress of bioinspired scalephobic surfaces with specific barrier layers. *Langmuir* **37**, 8639–8657 (2021).

18. G. Azimi, Y. Cui, A. Sabanska, K. K. Varanasi, Scale-resistant surfaces: Fundamental studies of the effect of surface energy on reducing scale formation. *Appl. Surf. Sci.* **313**, 591–599 (2014).

19. H. Müller-Steinhagen, Q. Zhao, Investigation of low fouling surface alloys made by ion implantation technology. *Chem. Eng. Sci.* **52**, 3321–3332 (1997).

20. H. Sojoudi, S. K. Nemani, K. M. Mullin, M. G. Wilson, H. Aladwani, H. Lababidi, K. K. Gleason, Micro-/nanoscale approach for studying scale formation and developing scale-resistant surfaces. *ACS Appl. Mater. Interfaces* **11**, 7330–7337 (2019).

21. J. Zhao, M. Wang, M. S. Jebutu, M. Zhu, K. K. Gleason, Fundamental nanoscale surface strategies for robustly controlling heterogeneous nucleation of calcium carbonate. *J. Mater. Chem. A Mater.* **7**, 17242–17247 (2019).

22. H. U. Zettler, M. Wei, Q. Zhao, H. Müller-Steinhagen, Influence of surface properties and characteristics on fouling in plate heat exchangers. *Heat Transfer Eng.* **26**, 3–17 (2005).

23. S. A. McBride, H.-L. Girard, K. K. Varanasi, Crystal critters: Self-ejection of crystals from heated, superhydrophobic surfaces. *Sci. Adv.* **7**, eabe6960 (2021).

24. W. Jiang, J. He, F. Xiao, S. Yuan, H. Lu, B. Liang, Preparation and antiscaling application of superhydrophobic anodized CuO nanowire surfaces. *Ind. Eng. Chem. Res.* **54**, 6874–6883 (2015).

25. S. B. Subramanyam, G. Azimi, K. K. Varanasi, Designing lubricant-impregnated textured surfaces to resist scale formation. *Adv. Mater. Interfaces* **1**, 1300068 (2014).

26. X. Yao, W. Lin, M. Wang, S. Wang, Nature-inspired high temperature scale-resistant slippery lubricant-induced porous surfaces (HTS-SLIPS). *Small* **18**, e2203615 (2022).

27. Z. Liu, C. Zhang, J. Jing, X. Zhang, C. Wang, F. Liu, M. Jiang, H. Wang, Bristle worm inspired ultra-durable superhydrophobic coating with repairable microstructures and anti-corrosion/scaling properties. *Chem. Eng. J.* **436**, 135273 (2022).

28. M. F. B. Sousa, H. C. Loureiro, C. A. Bertran, Anti-scaling performance of slippery liquid-infused porous surface (SLIPS) produced onto electrochemically-textured 1020 carbon steel. *Surf. Coat Technol.* **382**, 125160 (2020).

29. S. Jo, H. Lee, H. Jang, D. R. Kim, Controlled integration of interconnected pores under polymeric surfaces for low adhesion and antiscaling performance. *ACS Appl. Mater. Interfaces* **13**, 13684–13692 (2021).

30. J. E. Friis, G. Subbiahdoss, G. Gerved, A. H. Holm, O. Santos, A. B. Blichfeld, S. Z. Moghaddam, E. Thormann, K. Daasbjerg, J. Iruthayaraj, R. L. Meyer, Evaluation of surface-initiated polymer brush as anti-scaling coating for plate heat exchangers. *Prog. Org. Coat.* **136**, 105196 (2019).

31. Y. Chen, X. Yu, L. Chen, S. Liu, X. Xu, S. Zhao, S. Huang, X. Tian, Dynamic poly(dimethylsiloxane) brush coating shows even better antiscaling capability than the low-surface-Energy fluorocarbon counterpart. *Environ. Sci. Technol.* **55**, 8839–8847 (2021).

32. A. Masoudi, P. Irajizad, N. Farokhnia, V. Kashyap, H. Ghasemi, Antiscaling magnetic slippery surfaces. *ACS Appl. Mater. Interfaces* **9**, 21025–21033 (2017).

33. T. Zhang, Y. Wang, F. Zhang, X. Chen, G. Hu, J. Meng, S. Wang, Bio-inspired superhydrophilic coatings with high anti-adhesion against mineral scales. *NPG Asia Mater.* **10**, e471 (2018).

34. H. Zhao, S. Khodakarami, C. A. Deshpande, J. Ma, Q. Wu, S. Sett, N. Miljkovic, Scalable slippery omniphobic covalently attached liquid coatings for flow fouling reduction. *ACS Appl. Mater. Interfaces* **13**, 38666–38679 (2021).

35. M. Mayer, W. Augustin, S. Scholl, Adhesion of single crystals on modified surfaces in crystallization fouling. *J. Cryst. Growth* **361**, 152–158 (2012).

36. W. C. Cheong, P. H. Gaskell, A. Neville, Substrate effect on surface adhesion/crystallisation of calcium carbonate. *J. Cryst. Growth* **363**, 7–21 (2013).

37. K. Al-Anezi, D. J. Johnson, N. Hilal, An atomic force microscope study of calcium carbonate adhesion to desalination process equipment: Effect of anti-scale agent. *Desalination* **220**, 359–370 (2008).

38. R. Zhang, G. Azimi, Scale-phobic surfaces made of rare earth oxide ceramics. *ACS Appl. Mater. Interfaces* **12**, 42339–42347 (2020).

39. R. Yang, K. K. Gleason, Ultrathin antifouling coatings with stable surface zwitterionic functionality by initiated chemical vapor deposition (iCVD). *Langmuir* **28**, 12266–12274 (2012).

40. N. Abdel-Aal, K. Satoh, K. Sawada, Study of the adhesion mechanism of CaCO3 using a combined bulk chemistry/QCM technique. *J. Cryst. Growth* **245**, 87–100 (2002).

41. L.-C. Wang, S.-F. Li, L.-B. Wang, K. Cui, Q.-L. Zhang, H.-B. Liu, G. Li, Relationships between the characteristics of CaCO 3 fouling and the flow velocity in smooth tube. *Exp. Therm. Fluid. Sci.* **74**, 143–159 (2016).

42. J. Y. M. Chew, W. R. Paterson, D. I. Wilson, V. Höufling, W. Augustin, A method for measuring the strength of scale deposits on heat transfer surfaces. *Dev Chem. Eng. Mineral Proc.* **13**, 21–30 (2005).

43. I. A. Løge, B. U. Anabaraonye, P. L. Fosbøl, Growth mechanisms of composite fouling: The impact of substrates on detachment processes. *Chem. Eng. J.* **446**, 137008 (2022).

44. M. Liu, S. Wang, L. Jiang, Nature-inspired superwettability systems. *Nat. Rev. Mater.* **2**, 17036 (2017).

45. S. Feng, P. Zhu, H. Zheng, H. Zhan, C. Chen, J. Li, L. Wang, X. Yao, Y. Liu, Z. Wang, Three-dimensional capillary ratchet-induced liquid directional steering. *Science* **373**, 1344–1348 (2021).

46. P. Papadopoulos, B. El Pinchasik, M. Tress, D. Vollmer, M. Kappl, H. J. Butt, Wetting of soft superhydrophobic micropillar arrays. *Soft Matter* **14**, 7429–7434 (2018).

47. K. L. Johnson, K. Kendall, A. D. Roberts, Surface energy and the contact of elastic solids. *Proc. R. Soc. Lond. A* **324**, 301–313 (1971).

48. R. W. Style, A. Jagota, C.-Y. Hui, E. R. Dufresne, Elastocapillarity: Surface tension and the mechanics of soft solids. *Annu. Rev. Condens. Matter. Phys.* **8**, 99–118 (2017).

49. K. R. Shull, Contact mechanics and the adhesion of soft solids. *Mater. Sci. Eng. R* **36**, 1–45 (2002).

50. N. Epstein, Particulate fouling of heat transfer surfaces: Mechanisms and models, in *Fouling Science and Technology* (Springer Netherlands, 1988), pp. 143–164.

51. Y. Xiang, S. Huang, P. Lv, Y. Xue, Q. Su, H. Duan, Ultimate stable underwater superhydrophobic state. *Phys. Rev. Lett.* **119**, 134501 (2017).

52. G. M. Burdick, N. S. Berman, S. P. Beaudoin, Describing hydrodynamic particle removal from surfaces using the particle Reynolds number. *J. Nanopart. Res.* **3**, 453–465 (2001).

53. Y. Jiang, S. Matsusaka, H. Masuda, Y. Qian, Characterizing the effect of substrate surface roughness on particle-wall interaction with the airflow method. *Powder Technol.* **186**, 199–205 (2008).

54. M. K. Chaudhury, K. H. Kim, Shear-induced adhesive failure of a rigid slab in contact with a thin confined film. *Eur. Phys. J. E Soft Matter.* **23**, 175–183 (2007).

55. D. L. Beemer, W. Wang, A. K. Kota, Durable gels with ultra-low adhesion to ice. *J. Mater. Chem. A Mater.* **4**, 18253–18258 (2016).

56. C. Wang, T. Fuller, W. Zhang, K. J. Wynne, Thickness dependence of ice removal stress for a polydimethylsiloxane nanocomposite: Sylgard 184. *Langmuir* **30**, 12819–12826 (2014).

57. Y.-W. Lin, T. Li, Y. Zhang, W.-W. Yan, X.-M. Chen, Z.-S. Zhang, J.-Y. Wu, Adhesion strength of tetrahydrofuran hydrates is dictated by substrate stiffness. *Pet. Sci.* 10.1016/j.petsci.2023.08.009 (2023).

58. A. Dhyani, J. Wang, A. K. Halvey, B. Macdonald, G. Mehta, A. Tuteja, Design and applications of surfaces that control the accretion of matter. *Science* **373**, eaba5010 (2021).

59. R. W. Style, C. Hyland, R. Boltyanskiy, J. S. Wettlaufer, E. R. Dufresne, Surface tension and contact with soft elastic solids. *Nat. Commun.* **4**, 2728 (2013).

60. R. W. Style, R. Boltyanskiy, Y. Che, J. S. Wettlaufer, L. A. Wilen, E. R. Dufresne, Universal deformation of soft substrates near a Contact line and the direct measurement of solid surface stresses. *Phys. Rev. Lett.* **110**, 066103 (2013).

61. G. Ziskind, M. Fichman, C. Gutfinger, Adhesion moment model for estimating particle detachment from a surface. *J. Aerosol Sci.* **28**, 623–634 (1997).

62. Y. Lai, D. He, Y. Hu, Indentation adhesion of hydrogels over a wide range of length and time scales. *Extrem. Mech. Lett.* **31**, 100540 (2019).

63. F. Yang, Adhesive contact between a rigid axisymmetric indenter and an incompressible elastic thin film. *J. Phys. D Appl. Phys.* **35**, 2614–2620 (2002).

64. M. M. Sharma, H. Chamoun, D. S. H. S. R. Sarma, R. S. Schechter, Factors controlling the hydrodynamic detachment of particles from surfaces. *J. Colloid Interface Sci.* **149**, 121–134 (1992).

65. M. E. O'Neill, A sphere in contact with a plane wall in a slow linear shear flow. *Chem. Eng. Sci.* **23**, 1293–1298 (1968).

66. A. M. Brzozowska, S. Maassen, Rubayn Goh Zhi Rong, P. I. Benke, C.-S. Lim, E. M. Marzinelli, D. Jańczewski, Serena Lay-Ming Teo, G Julius Vancso, Effect of variations in micropatterns and surface modulus on marine fouling of engineering polymers. *ACS Appl. Mater. Interfaces* **9**, 17508–17516 (2017).

67. G. Ahmadi, Mechanics of particle adhesion and removal, in *Particle Adhesion and Removal*, K. L. Mittal, R. Jaiswal, Eds. (John Wiley & Sons, Inc., 2015), pp. 81–104.

68. A. C. Sagle, H. Ju, B. D. Freeman, M. M. Sharma, PEG-based hydrogel membrane coatings. *Polymer* **50**, 756–766 (2009).

69. H. Ju, B. D. McCloskey, A. C. Sagle, Y.-H. Wu, V. A. Kusuma, B. D. Freeman, Crosslinked poly(ethylene oxide) fouling resistant coating materials for oil/water separation. *J. Memb. Sci.* **307**, 260–267 (2008).

70. Y.-H. Wu, H. B. Park, T. Kai, B. D. Freeman, D. S. Kalika, Water uptake, transport and structure characterization in poly(ethylene glycol) diacrylate hydrogels. *J. Memb. Sci.* **347**, 197–208 (2010).

71. F. Della Sala, M. Biondi, D. Guarnieri, A. Borzacchiello, L. Ambrosio, L. Mayol, Mechanical behavior of bioactive poly(ethylene glycol) diacrylate matrices for biomedical application. *J. Mech. Behav. Biomed. Mater.* **110**, 103885 (2020).

72. H. Martin, *Heat Exchangers* (Routledge, ed. 1, 2018).

73. R. Mukherjee, Effectively design shell-and-tube heat exchangers. *Chem. Eng. Prog.* **94**, 21–37 (1998).

74. F. C. Hsia, S. Franklin, P. Audebert, A. M. Brouwer, D. Bonn, B. Weber, Rougher is more slippery: How adhesive friction decreases with increasing surface roughness due to the suppression of capillary adhesion. *Phys. Rev. Res.* **3**, 43204 (2021).

75. M. J. Hokkanen, M. Backholm, M. Vuckovac, Q. Zhou, R. H. A. Ras, Force-based wetting characterization of stochastic superhydrophobic coatings at nanonewton sensitivity. *Adv. Mater.* **33**, e2105130 (2021).

76. S. Mishima, T. Ougizawa, Microsphere adhesion on rubber films accompanied by sphere sedimentation. *Langmuir* **36**, 6597–6604 (2020).

77. R. Hopf, L. Bernardi, J. Menze, M. Zündel, E. Mazza, A. E. Ehret, Experimental and theoretical analyses of the age-dependent large-strain behavior of Sylgard 184 (10:1) silicone elastomer. *J. Mech. Behav. Biomed. Mater.* **60**, 425–437 (2016).

78. P. D. Garcia, R. Garcia, Determination of the elastic moduli of a single cell cultured on a Rigid support by force microscopy. *Biophys. J.* **114**, 2923–2932 (2018).

79. M. H. Nielsen, S. Aloni, J. J. De Yoreo, In situ TEM imaging of CaCO3 nucleation reveals coexistence of direct and indirect pathways. *Science* **345**, 1158–1162 (2014).

80. D. Ershov, M.-S. Phan, J. W. Pylvänäinen, S. U. Rigaud, L. Le Blanc, A. Charles-Orszag, J. R. W. Conway, R. F. Laine, N. H. Roy, D. Bonazzi, G. Duménil, G. Jacquemet, J.-Y. Tinevez, TrackMate 7: Integrating state-of-the-art segmentation algorithms into tracking pipelines. *Nat. Methods.* **19**, 829–832 (2022).

81. Paul Marienfeld GmbH & Co. KG, "Chemical and physical properties Microscope slides made of soda lime glass of the 3rd hydrolytic class" (Lauda-Königshofen, 2020).

82. T. R. Tuladhar, W. R. Paterson, N. Macleod, D. I. Wilson, Development of a novel non-contact proximity gauge for thickness measurement of soft deposits and its application in fouling studies. *Can. J. Chem. Eng.* **78**, 935–947 (2000).

83. P. W. Gordon, A. D. M. Brooker, Y. M. J. Chew, D. I. Wilson, D. W. York, A scanning fluid dynamic gauging technique for probing surface layers. *Meas. Sci. Technol.* **21**, 085103 (2010).
